# A new cuspidate ptychodontid shark (Chondrichthyes; Elasmobranchii), from the Upper Cretaceous of Morocco with comments on tooth functionalities and replacement patterns

**DOI:** 10.1016/j.jafrearsci.2021.104440

**Published:** 2021-12-21

**Authors:** Manuel Amadori, René Kindlimann, Eliana Fornaciari, Luca Giusberti, Jürgen Kriwet

**Affiliations:** a Department of Palaeontology, Geozentrum, University of Wien, Althanstra βe 14, 1090, Wien, Austria; b Sammlung R. Kindlimann, Aathal-Seegräben, Switzerland; c Dipartimento di Geoscienze, Universita degli Studi di Padova, Via Gradenigo 6, I-35131, Padova, Italy

**Keywords:** Dental plate, Heterodonty, Durophagy, Taxonomy, Asfla member, Goulmima region

## Abstract

The first articulated dentition of †*Ptychodus* from Africa is described herein. The specimen, likely coming from the Turonian of the Asfla area (Goulmima region, southeastern Morocco), exhibits a well-preserved lower dental plate of a second-level predator. A new species, †*P. maghrebianus* sp. nov., is erected herein based on this durophagous dentition characterised by imbricated cuspidate teeth. We employed for the first time in †*Ptychodus* multiple quantitative analyses and statistical parametric and non-parametric tests to process biometrical data taken from articulated, associated and isolated teeth. The quantitative approach (morphospace analysis) is exploited herein to support the traditional taxonomic identification (qualitative examination) of †*P. maghrebianus* sp. nov. and to separate it from the similar cuspidate species, †*P. mortoni*. Morphospace reconstructions confirm a marked lower dental heterodonty (mesio-distal patterns) for both species. The analysis protocol employed here also allows assigning indeterminate teeth as belonging to †*P. mortoni*. The reconstruction of the entire lower dental plate of †*P. maghrebianus* sp. nov. shows a cuspidate dentition probably able to reduce tooth damages when crushing thin-shelled prey. Both dental morphologies and tooth wear patterns suggest a peculiar food processing and a diet mainly consisting of bivalves, decapods and small fish for this durophagous predator. Trophic reconstructions of the Turonian ichthyofauna inhabiting the middle to outer ramp environment of the Asfla area emphasize that †*P. maghrebianus* sp. nov. and the batoid †*Tingitanius* most likely represented second-level consumers, whereas the sclerorhynchiforms †*Asflapristis* and †*Ptychotrygon* represented third-level predators. Top positions within the food web were occupied by larger predaceous elasmobranchs (e.g., †*Squalicorax*).

## 1 Introduction

Vertebrate remains are very abundant in Cretaceous deposits of southern and central Morocco (e.g. Cavin et al., 2001, 2010; Angst and Bardet, 2015; Vullo et al., 2016, 2017; Allemand et al., 2018). In this region, excavation activities by local people have particularly intensified during the past two decades to collect Cretaceous fossils for commercial purposes, but although numerous finds were discovered by these commercial activities, associated data on the original sedimentology, stratigraphy and provenience of the specimens, however, often are lacking (Cavin et al., 2010). Moreover, remains are commonly unearthed incompletely and damaged mainly due to the rudimentary techniques used by local collectors to extract fossils from the rocks. Moreover, fragmentary fossils with no commercial value are often neglected and discarded, resulting in a dramatic loss from a scientific perspective (Mcgowan and Dyke, 2009). Despite the countless number of discarded finds, elasmobranch (*sensu* Maisey, 2012) remains in various degrees of preservations, are widely documented from Upper Cretaceous deposits of various Moroccan localities (e.g. Cappetta, 1987; Dutheil and Ackermann, 2004; Cavin et al., 2010; Claeson et al., 2013; Cappetta et al., 2014; Vullo et al., 2016; Villalobos-Segura et al., 2019). For example, a highly diversified elasmobranch fauna, consisting of about 60 taxa and including both sharks and rays, was reported from the Maastrichtian of Benguérir (Ganntour Basin, southwestern Morocco; see Cappetta et al., 2014). Marine and freshwater bony fishes, as well as elasmobranch and hybodont remains (e.g. teeth and fin spines), are also common in the famous ‘mid’-Cretaceous fossil beds of Kem Kem (southeastern Morocco; see Dutheil, 1999; Cavin et al., 2010, 2015). Among the most well-preserved specimens, Vullo et al. (2016) documented an articulated mackerel shark (Lamniformes) from the upper Cenomanian -?lower Turonian Lagerstätte of Agoult (Akrabou Formation, Kem Kem region, southeastern Morocco). Additionally, three-dimensionally preserved skeletal remains of batoids (e.g. platyrhinid and sclerorhynchid) were described from the Turonian of the Goulmima region (Errachidia Province, southeastern Morocco; see Claeson et al., 2013; Villalobos-Segura et al., 2019; Cooper and Martill, 2020). Dental remains and other unspecified material assignable to the extinct elasmobranch genus †*Ptychodus* were briefly reported from the Upper Cretaceous of northern and western Morocco (see also ‘Moroccan record of †*Ptychodus*’, below).

The extinct shark †*Ptychodus* primarily is known by its peculiar molariform teeth and, more rarely, cranial and postcranial skeletal remains such as, e.g. vertebral centra, portions of mineralized cartilages and placoid scales (e.g. Everhart and Caggiano, 2004; Shimada et al., 2009, 2010; Amadori et al., 2020a; Jambura and Kriwet, 2020). †*Ptychodus* was a ubiquitous elasmobranch, as its associated and isolated teeth are widely reported from the Upper Cretaceous of Africa, Asia, Europe and North and South America (e.g. Antunes and Cappetta, 2002; Dutheil and Ackermann, 2004; Everhart and Caggiano, 2004; Cappetta, 2012; Shimada, 2012; Diedrich, 2013; Vullo and Courville, 2014; Hamm, 2017, 2019, 2020; Amadori et al., 2019b, 2020a, b). The dentition of †*Ptychodus* consists of teeth that are arranged in antero-posteriorly directed dental rows to form maxillary and mandibular tooth plates exhibiting different degrees of dignathic heterodonty (see Woodward, 1887, 1904, 1912; Williston, 1900a, b; Shimada, 2012; Amadori et al., 2019b, 2020a). The marked interspecific and intraspecific morphological variabilities displayed by the dentitions of †*Ptychodus* often complicate its taxonomic identification and systematic position to and within elasmobranchs (Nicholls, 2010). Nevertheless, a recent histological-based study considers this enigmatic predator as closely related to elasmobranchs rather than extinct hybodontiform sharks (see Hoffman et al., 2016).

Within †*Ptychodus*, two different morphological groups comprising taxa with distinct cuspidate and low-crowned teeth, respectively, can easily be separated (Amadori et al., 2019b, 2020a). †*Ptychodus* most likely was a durophagous predator able to crush hard-shelled prey with its pavement-like dentitions (e.g. Williston, 1900a, b; Fossa-Mancini, 1921; Kauffman, 1972; Shimada, 2012; Everhart, 2017; Hamm, 2017, 2019, 2020; Amadori et al., 2020a, b). Opportunistic feeding also was hypothesized for some cuspidate species largely preying on soft-bodied molluscs, crustaceans, and small fishes (e.g. Shimada et al., 2009; Amadori et al., 2019b, 2020b).

Here, we report the first, still articulated lower dental plate consisting of characteristic cuspidate teeth of a hitherto unknown †*Ptychodus* species, †*P. maghrebianus* sp. nov., from the Turonian (Upper Cretaceous) of Morocco and discuss its feeding specialization and diet preferences. Qualitative and quantitative approaches are employed herein to compare the morphospaces of the new taxon to the morphologically most-similar species, †*P. mortoni*. Both intraspecific (e.g. mesio-distal heterodonty) and interspecific variabilities of each taxon are analysed in the present study. Indeterminate isolated teeth also are taxonomically identified here employing the same approach. The Turonian marine trophic web for the Asfla area, is reconstructed based on a review of published data and the dental morphology of †*P. maghrebianus* sp. nov. New insights into the distribution patterns of †*Ptychodus* in Morocco additionally are provided based on our results.

## 2 Moroccan record of y*Ptychodus*

**Fig. 1:**
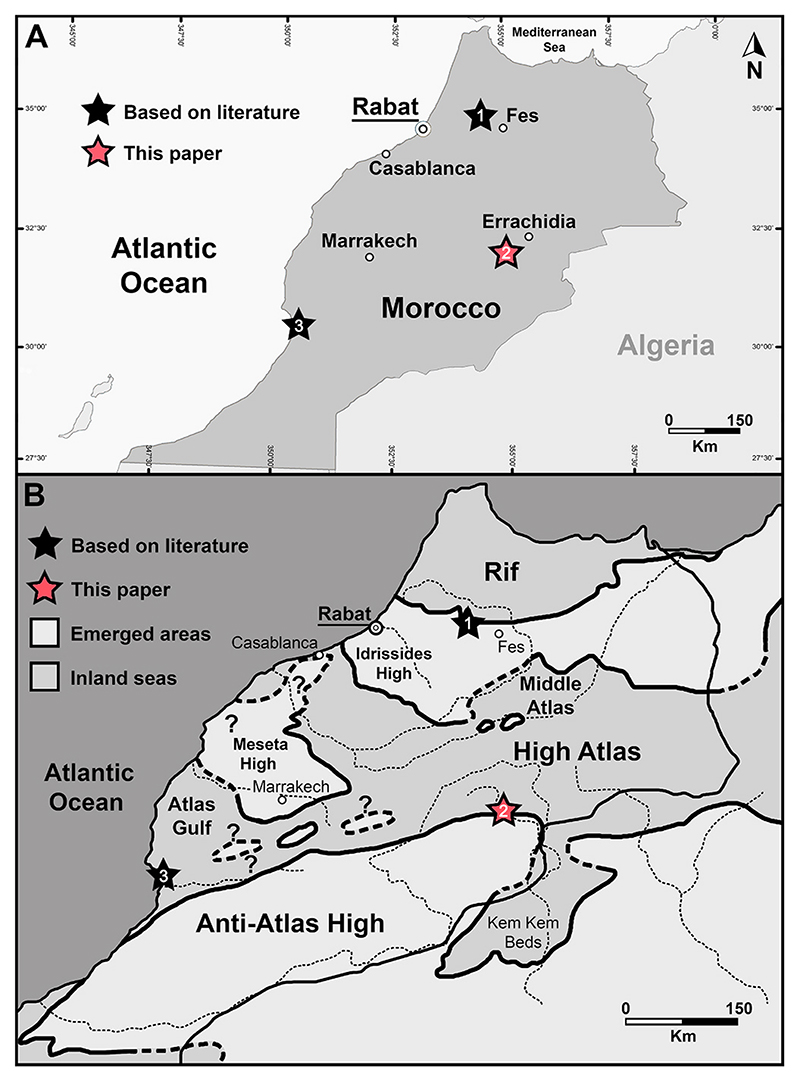
Location of the Moroccan fossiliferous sites (A) yielding †*Ptychodus* remains (stars) and palaeogeographical scenario map (B) of the Cenomanian-Turonian transgression in Morocco (modified after Bardet et al., 2003a). 1, Jbel Tselfat area; 2, Goulmima region; 3, Agadir region.

Cappetta (1987, p. 38) reported the genus †*Ptychodus* for the first time from the Upper Cretaceous of the Agadir region (western Morocco; see Fig. 1A) without further information on the provenance or the nature of the finds (see also Cappetta, 2012). From south to north, basinal sedimentary rocks of Tarfaya, Agadir, Essaouira, as well as at the margin of the Meseta, represent the preserved Cretaceous depositional area of the Atlantic Moroccan margin (see Wiedmann et al., 1982) with Upper Cretaceous deposits almost exclusively cropping out in the northern and western surroundings of the Agadir area (see Saadi et al., 1985; Garassino et al., 2011; Jaillard et al., 2019). Turonian deposits within the Agadir basin (‘Atlas Gulf’; see Fig. 1B) mainly consist of bituminous marls (black shales) characterized by carbonaceous laminated marls with intercalated pelagic limestones and cherts, which were deposited in a sea depth of 200–300 m (upper bathyal; Einsele and Wiedmann, 1982; Wiedmann et al., 1982).

Dutheil and Ackermann (2004) reported additional small teeth of †*Ptychodus* (?*P. decurrens*) testified by dental imprints from the upper Cenomanian of Jbel Tselfat (or ‘Djebel Tselfat’, northwestern Morocco; see Fig. 1A). The Cenomanian-Turonian black shale deposits outcropping in this area (e.g. Aïn el Kerma and Sigda) indicate a poorly oxygenated deep-sea environment (probably deeper than 500 m) deposited under a hot and humid climate and possibly associated with the Oceanic Anoxic Event 2 (OAE2; see Khalloufi et al., 2010; Ward et al., 2014). The Upper Cretaceous deposits of Jbel Tselfat have yielded a rich and well-diversified Cretaceous ichthyofauna up to now, whose only chondrichthyan representative, however, is †*Ptychodus* (Arambourg, 1954; Rindone, 2008; Khalloufi et al., 2010; Ward et al., 2014).

## 3 Geological setting

**Fig. 2:**
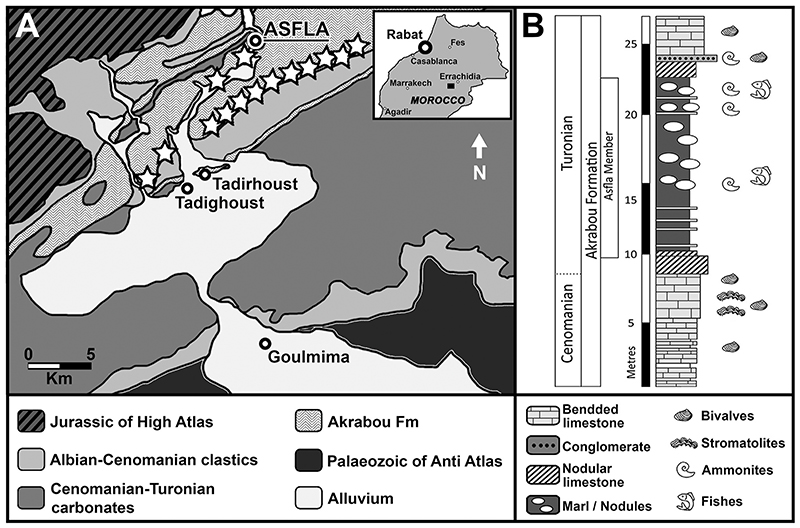
Locality map (A; white stars indicate the main fossil quarries) and stratigraphic column (B) of the Akrabou Fm in the surrounding of Asfla area (Goulmima area, southeastern Morocco). Modified after Claeson et al. (2013) and according to Saadi et al. (1985), Villalobos-Segura et al. (2019) and Cooper and Martill (2020).

The well recognizable cliff ‘Hamada’ cropping out extensively from the Gulf of Agadir (southwestern Morocco) to the Gulf of Gabes (eastern Tunisia) represents the main ‘mid’-Cretaceous (Aptian–Turonian) deposits of northwestern Africa (Choubert and Faure-Muret, 1962; Angst and Bardet, 2015). From the base to the top, the series exposed at Hamada classically include three formations: (1) the red sandstones of the Ifezouane Fm., which are questionably referred to the Albian, (2) the Albian-Cenomanian Aoufous Fm, mainly consisting of green marls and lagunar gypsous marly sandstones, and (3) the limestone of Akrabou Fm representing the beginning of the Cenomanian-Turonian transgression in the area (see also Ettachfini and Andreu, 2004; Cavin et al., 2010; Angst and Bardet, 2015). The Hamada domain, belonging to the Preafrican platform, comprises the narrow Imini and Goulmima plateaus that are wedged between the Anti- and the High-Atlas (Ferrandini et al., 1985; Ettachfini and Andreu, 2004; Zouhri et al., 2008; Cavin et al., 2010). The Cretaceous succession cropping out in the surroundings of the Goulmima area (Fig. 2A) overlies the folded Palaeozoic basement of the Anti-Atlas, forming a foreland to the folded successions of the High-Atlas in the north (Claeson et al., 2013). The lower part of the Cretaceous succession mainly consists of non-marine Albian–Cenomanian deposits (Ifezouane and Aoufous formations), overlaid by upper Cenomanian-Turonian shallow-water carbonates (Akrabou Formation; Ettachfini and Andreu, 2004). The Akrabou Formation also includes marly limestones, marls, and other impure micrites with ovoid calcareous concretions (see “Asfla Member” in Fig. 2B) testifying to middle to outer ramp early Turonian marine environment (e.g. Lézin et al., 2012; Meister et al., 2017; Villalobos-Segura et al., 2019; Cooper and Martill, 2020; Veysey et al., 2020).

Local people commonly excavate various localities in the Goulmima area (e.g. the villages of Tadirhoust and Asfla; see white asterisk in Fig. 2), searching for macrofossils, such as exceptionally preserved vertebrate remains usually embedded in calcareous nodules (e.g. Bardet et al., 2003a, b; Cavin et al., 2010; Claeson et al., 2013; Angst and Bardet, 2015; Allemand et al., 2018; Cooper and Martill, 2020; Villalobos-Segura et al., 2021). In particular, a sequence of alternating facies succession consisting of fossiliferous packstones, chalky marls, cherts and vertebrate-bearing nodular horizons belonging to the Asfla Member (Fig. 2B) of the Akrabou Fm of the Asfla area are targeted for vertebrate fossils (e.g. Cavin et al., 2010; Claeson et al., 2013; Angst and Bardet, 2015; Villalobos-Segura et al., 2019; Cooper and Martill, 2020). The Asfla Member is assigned to the early Turonian based on its ammonite fauna (*Mammites nodosoides* Zone; Meister et al., 2017; Kennedy et al., 2008; Cavin et al., 2010). The macrofossil assemblages from the Goulmima area usually comprise ammonites, teleosts, pycnodont actino-pterygians, marine reptiles (e.g. mosasauroids and plesiosaurs) and, more rarely, elasmobranchs (e.g. Bardet et al., 2003a, b; Kennedy et al., 2008; Cavin et al., 2010; Claeson et al., 2013; Angst and Bardet, 2015; Gale et al., 2017; Meister et al., 2017; Allemand et al., 2018; Villalobos-Segura et al., 2019, 2021; Cooper and Martill, 2020). Additionally, the limestones of the Akrabou Fm include shell beds, with a low-diversified bivalve assemblage, and stromatolites in various horizons (Kennedy et al., 2008; Cooper and Martill, 2020).

The articulated tooth set presented here is preserved in a light brown calcareous ‘concretion’ (nodule) and it undoubtedly belongs to a single individual. The preservation and matrix texture of this specimen are consistent with that of the main fossil-bearing horizon of the Goulmima region (Errachidia Province; see also Claeson et al., 2010b, 2013). In this area, the shape and size of the nodules can greatly vary, sometimes completely embedding the fossil remains or only partially incorporating them. Additionally, fossils from this region also might be contained in more or less continuous limestone layers in certain levels (L. Cavin, pers. comm. 2021). ‘Concretions’ identical to that enclosing the specimen described here are easily recognizable around vertebrate fossils from the Turonian of Asfla and Tadighoust villages (Goulmima region, L. Cavin, pers. comm. 2021).

## 4 Materials and methods

### 4.1 Materials

The articulated dentition described herein, originally purchased by one of us (RK) at the Munich Mineralshow in 2015, is part of the collection of the Palaeontological Institute and Museum of the University of Zurich, Switzerland (catalogue number: PIMUZ A/I 5056). The specimen is currently on display in the ‘Haimuseum und Sammlung R. Kindlimann’ in Aathal-Seegräben (Switzerland), with public access. The tooth labelled here ‘PIMUZ A/I 5056α’ originally was part of specimen PIMUZ A/I 5056 but subsequently was detached. Specimens used for comparative purposes include the holotype of the cuspidate species, †*Ptychodus mortoni* (NHMUK OR 28394; see also Everhart, 2013: Fig. 3) and five articulated and associated tooth sets, respectively, which are assigned to the same species here. The articulated sets consist of three lower dentitions (KUVP 119675, ROM 21705 and UNSM 1194), which were extensively documented by Shimada (2012: Fig. 3E,4, 5), an associated tooth set (FHSM VP-335; see Hamm, 2020: text fig. 99), and a lower symphyseal tooth (here labelled ‘FHSM VP-17415C’) associated with a large portion of the Meckel’s cartilage of specimen FHSM VP-17415 (see Shimada et al., 2010: Fig. 2C). Additionally, three isolated and still indeterminate teeth, MT-G-011, MT-G-012, and MT-G-013 (see Carrillo, 2009: fig.3.1a-3.3b) exhibiting dental morphologies similar to those typical for †*P. mortoni* were included in the analyses here. See Table 1, for details of the specimens.

### 4.2 Methods

#### 4.2.1 General procedures

**Fig. 3:**
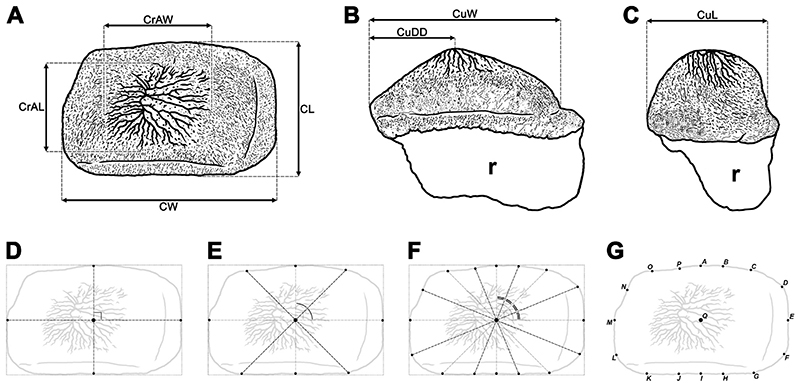
Morphometric tooth terminology. The linear measurements collected in occlusal (A), posterior (B) and lateral (C) views include transversal width of the crown (CW), anteroposterior length of the crown (CL), width of the cusp (CuW), length of the cusp (CuL), distal displacement of the cusp (CuDD), transversal width of the crested area (CrAW) and anteroposterior length of the crested area (CrAL). In occlusal view (D–G), additional linear measurements have been taken between the “center of the tooth crown” (point “*Q*”) and 16 “biometric marks” (points “*A*-*P*”).

Two photographic cameras, Olympus E-MD5 and Olympus E-MD1 Mark II with a mounting macro lens Olympus M. Zuiko Digital ED Objektiv (12–50 mm) were used to create high-quality photos of all specimens. The illustrative drawings and images of the fossils were prepared using the software packages GIMP (v. 2.8.16) and Photoshop CS5 (v.12.0 × 32). A smear slide of the matrix embedding the new specimen was prepared for calcareous nannofossil analysis to obtain an exact stratigraphic age of the specimen (see Amadori et al., 2019b, 2020a for detailed information on the procedure). The anatomical and odontological terminologies used here for †*Ptychodus* follow Cappetta (2012), Shimada (2012) and Amadori et al. (2020a). For the morphometric tooth terminology, see Fig. 3.

#### 4.2.2 Multiples analyses

A fragmentary fossil record largely composed of isolated teeth often hampers the dental-based taxonomic identification in elasmobranchs, which is even more complex for taxa without extant relatives (Shimada, 2005; Cappetta, 2012; Marramà and Kriwet, 2017; Amadori et al., 2020a). One of the major problems is represented by the impossibility of correctly evaluating the intraspecific variability of each group due to the widespread heterodonty in both sharks and rays (Cappetta, 2012; Marramà and Kriwet, 2017). Furthermore, qualitative tooth features (e. g. overall shape, ornamentations and presence of folds or cusps) are predominately employed for the identification of fossil species in sharks with rare exceptions (e.g. Adnet, 2006; Cappetta, 2012; Marramà and Kriwet, 2017; Fialho et al., 2018; Salama et al., 2020). However, it has been convincingly demonstrated that quantitative approaches (e.g. geometric morphometric and biometric characters), combined with traditional, qualitative investigations based on descriptive traits, can greatly enhance the reliability of taxonomic identifications in elasmobranchs, and in other vertebrates as well (e.g. Smith et al., 2005; Adnet, 2006; Whitenack and Gottfried, 2010; Brophy et al., 2014; Marramà and Kriwet, 2017; Galán et al., 2019; Salama et al., 2020). In this perspective, multivariate statistical techniques as principal component (PCA) and linear discriminant analyses (LDA) based on a large set of quantifiable variables (e.g. pure linear measurements) can be extremely useful to support heterodont pattern investigation and taxonomic identification in fossil shark employing a rigorous protocol and statistic tests for evaluating their effectiveness (e.g. Marramà and Kriwet, 2017; Cullen and Marshall, 2019).

##### 4.2.2.1 Taxon sampling

Articulated upper dental plates of †*Ptychodus maghrebianus* sp. nov. are unknown and only the lower dental plate, which nevertheless provides sufficient important characters for defining a new species, was recovered. Consequently, the statistical analyses presented herein focus only on lower dentitions for a rigorous biometric comparison and the articulated dentitions available for the present study thus ensure a detailed evaluation of the interspecific variability. This helps limiting taxonomic overestimations, which results from the prevalent dental heterodonties in †*Ptychodus* species. The material used for the analyses includes the articulated specimen of †*P. maghrebianus* sp. nov. described herein as well as five articulated or associated dentitions of †*P. mortoni* to ensure an accurate comparison. Teeth assigned to subadult or juvenile forms were excluded in the present study (e.g. Hamm, 2020, text Figs. 97 and 98). Although the two homodont dentitions assigned to †*P. cyclodontis* by Brito and Janvier (2002) and Mutter et al. (2005) markedly differ from those of all other species within the family Ptychodontidae, a rigorous and careful reassessment of this material is mandatory to clarify the still ambiguous relationships between †*P. cyclodontis* and the other ptychodontids (see Shimada, 2012). Unfortunately, the necessary material for an accurate taxonomic review of this species is currently unavailable and †*P. cyclodontis* thus was excluded from present study. The taxonomic identification of three isolated teeth exhibiting a very acute cusp and radial occlusal ornamentations and previously referred to †*Ptychodus* sp. is also here included (see Table 1 for more details).

##### 4.2.2.2 Analytical protocol

Twenty-three linear measurements were assembled using the image analysis software Image J (v. 1.47) as depicted in Fig. 3. All measurements are in millimeters and they were approximated avoiding decimals (see ‘Data Archiving Statement’, below). The measurements collected include the transversal width of the crown (CW), the anteroposterior length of the crown (CL), the width of the cusp (CuW), the length of the cusp (CuL), the distal displacement of the cusp (CuDD), the transversal width of the crested area (CrAW), and the anteroposterior length of the crested area (CrAL). Since parts of the teeth are abraded, measurements of the height of the crown and of the cusp were not included. Additional measurements were taken between the ‘center of the tooth crown’ and 16 ‘biometric marks’ placed on the crown outline in occlusal view (Fig. 3D–G). The center of the quadrilateral circumscribed to the tooth outline was considered as crown center (points ‘Q’ in Fig. 3D–G); the base of the quadrilateral (bottom in Fig. 3D–G) is always tangent to the posterior side of the tooth crown. The first four ‘biometric marks’ were obtained by the intersection between the crown outline and the perpendicular lines crossing the center as depicted in Fig. 3D. Extending the bisectors of the angles from the center to the crown outline allowed to identify five additional ‘biometric marks’ (Fig. 3E), which was repeated twice (see also Fig. 3F). Measurements from ‘QB’ to ‘QH’ are always referred to the mesial tooth side, while those from ‘QJ’ to ‘QP’ are those taken on the distal side (see Fig. 3G). Measurements referred to the distal tooth edges have been taken on the left side of the symphyseal teeth. Measurements of the root were not taken, as the roots are rarely preserved or exposed in †*Ptychodus* specimens. Principal components analysis (PCA) based on the linear measurements (see ‘Data Archiving Statement’, below) was employed herein to investigate the intraspecific variability (e.g. mesiodistal patterns) and to provide reliable support for the taxonomic identification of specimens assignable to the cuspidate species †*P. maghrebianus* sp. nov. and †*P. mortoni*, respectively The PCA protocol of Marramà and Kriwet (2017) was employed or, if necessary, adapted for the analyses conducted herein. The logtransformation function was used as data treatment for overcoming the problem of the non-normal distribution of data by un-stretching large scales of values (see Smith et al., 2005; Hamm, 2010; Janzïekovic; and Novak, 2012; Marramà and Carnevale, 2015; Marramà and Kriwet, 2017), when articulated teeth derived from a single individual were analysed. Additionally, the proportion between each measurement (see above) and a standard ‘reference’ measurement (crown length; see CL in Fig. 3A) is employed herein to considerably reduce allometric size-dependent variations for comparisons between articulated or associated dentitions that belong to different individuals and possibly even to different developmental stages (Hammer, 2019). The similarities of in-group centroid positions and the degree of overlap between the different groups (†*P. maghrebianus* sp. nov., †*P. mortoni* and indeterminate specimens) were tested quantitatively through the multivariate analysis of variance (PERMANOVA) and the analysis of similarities (ANOSIM; see Clarke, 1993; Anderson, 2001; Hammer et al., 2001; Nyberg et al., 2010; Marramà and Kriwet, 2017; Cullen and Marshall, 2019; Hammer, 2019). The statistical robustness of the groups delineated in the PCA was tested herein through a linear discriminant analysis (LDA) or a canonical variate analysis (CVA) for comparisons between more than two groups (Sokal and Rohlf, 1995; Mclachlan, 2004; Buckley et al., 2010; Marramà and Kriwet, 2017; Galán et al., 2019; Hammer, 2019). The significance of each pairwise comparison was determined using the ‘Hotelling’s test’ (p-value < 0.05) and the ‘squared Mahalanobis distance’ (considerably different from 0), which is equivalent to Hotelling’s T-square distribution (see Kowalewski et al., 1997; Mclachlan, 2004; Marramà and Kriwet, 2017; Hammer, 2019). The pairwise squared distances between the centroids of the groups in the discriminant space (‘squared Mahalanobis distances’, significant when markedly higher than 0) was estimated using a parametric approach (MANOVA; Kowalewski et al., 1997; Smith et al., 2005; Hammer, 2019). Where necessary, results were adjusted using the ‘Bonferroni correction’ (multiplying by the number of pairwise comparisons) and/or ‘Jackknife resampling’ functions (deleting single observations from the original sample) in order to reduce any possible bias or spurious positives (Smith et al., 2005; Sahinler and Topuz, 2007; Hammer, 2019; Galán et al., 2019). The software package Past 3.25 was used for all the statistical analyses performed herein (see Hammer et al., 2001; Marramà and Kriwet, 2017; Galán et al., 2019; Hammer, 2019; Amadori et al., 2020b).

#### 4.2.3 Micropalaeontological analyses

Two small rock samples were removed from two different portions of the matrix embedding the specimen for micropalaeontological analyses. The rock samples were impregnated with Araldite®2029 Huntsman, then cut and polished using standard hard rock thin sectioning equipment and technique with minor modifications. Two 30-μm-thick thin sections of rock pasted with epoxy onto a glass slide were prepared for petrographic analysis using transmitted light optical microscopy. The remaining portions of the two samples were drilled with a millimetric tungsten carbide spherical drill bit mounted on an electric drill. The powder obtained was then utilized for preparing two smear slides for standard calcareous nannoplankton analyses.

#### 4.2.4 Institutional abbreviations

FHSM, Fort Hays State University’s Sternberg Museum of Natural History (Vertebrate Paleontology collection) in Hays (Kansas, USA); PIMUZ, Palaeontological Institute and Museum of the University of Zurich (Switzerland); KUVP, University of Kansas Museum of Natural History (Vertebrate Paleontology collection) in Lawrence (Kansas, U.S. A); MT-G, Colección de Geología y Paleontología del Museo Tulene Bertoni Trujillo (Trujillo, Venezuela); NHMUK, Natural History Museum in London (United Kingdom); ROM, Royal Ontario Museum in Toronto (Ontario, Canada); UNSM, University of Nebraska State Museum in Lincoln (Nebraska, USA).

## 5 Systematic palaeontology

This published work and the nomenclatural acts it contains have been registered with Zoobank: http://zoobank.org/References/680132ee-d7be-40c8-8c34-3398a13ca5a1

Class **Chondrichthyes** Huxley, 1880

Subclass **Elasmobranchii** Bonaparte, 1838

Order **yPtychodontiformes** Hamm, 2019

Family **yPtychodontidae** Jaekel, 1898

Genus **y*Ptychodus*** Agassiz, 1834

*Type species.* †*Ptychodus schlotheimii* Agassiz (1834) (nomen oblitum), senior synonym of †*Ptychodus latissimus* Agassiz (1835) (nomen protectum). See Brignon (2015) and Giusberti et al. (2018).

*Diagnosis*. See Woodward (1912) and Jambura and Kriwet (2020). **y*Ptychodus maghrebianus*** sp. nov.

Figs. 4-12

*LSID.*urn:lsid:zoobank.org:pub:680132EE-D7BE-40C8-8C34-3398A13CA5A1

*Holotype.* Specimen PIMUZ A/I 5056, an articulated portion of the lower dental plate.

*Type locality and horizon.* Goulmima region, southeastern Morocco. Micropalaeontological analyses conducted on the matrix were inconcludent for obtaining a biostratigraphic dating of the specimen. The two smear slides analysed for calcareous nannofossils were both completely barren, whereas the two thin sections revealed only the presence of scattered biserial small foraminifera, possible calcispheres along with indeterminable bioclasts and phosphatic grains. However, as previously stated, the characteristic preservation of the specimen in a large nodule of light brown limestone is highly compatible with a provenance of the fossil from the lower Turonian Asfla Member, which is the only fossiliferous interval in the Goulmima area yielding vertebrate remains preserved in calcareous nodules.

### Diagnosis

A species of †*Ptychodus* only known by the lower dental plate with the following dental features: tooth crowns rectangular and bulgy with a rounded and ticked cusp crossed by irregular and fine ridges radiating from the tooth central area. Each radial ridge subsequently branches basally into numerous thinning ridges gradually forming a dense, random granular pattern on the well-developed marginal areas, not following a diagnostic design. Cusp covers almost entire occlusal surface, extending to the tooth edges in symphyseal teeth. Mesial edge of lateral teeth displays an additional lowered area. Both anterior protuberance and posterior sulcus poorly developed. Root massive and bilobate, with shallow antero-posterior sulcus. Tooth outline becomes increasingly asymmetrical mesio-distally with a transversally elongated and parallelogram-shaped dental crown. Cusp almost completely reduced in distalmost teeth.

### Etymology

The specific epithet is derived from the geographical term ‘Maghrib’ used by Muslim Arabs for northern Africa extending from Alexandria to the Atlantic coast including Morocco, from where the specimen comes and the Latin suffix ‘-ianus’, indicating its origin from this region.

### Description

**Fig. 4:**
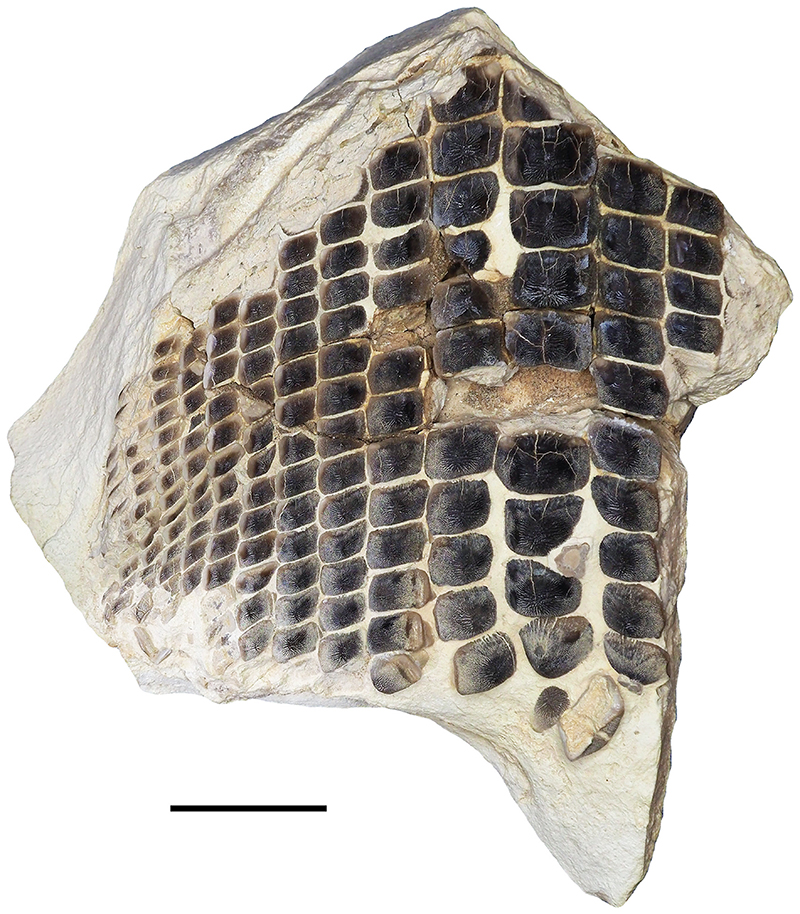
Holotype PIMUZ A/I 5056 of †*Ptychodus maghrebianus* sp. nov. coming from the Upper Cretaceous of Morocco. Scale bar equals 50 mm.

**Fig. 5:**
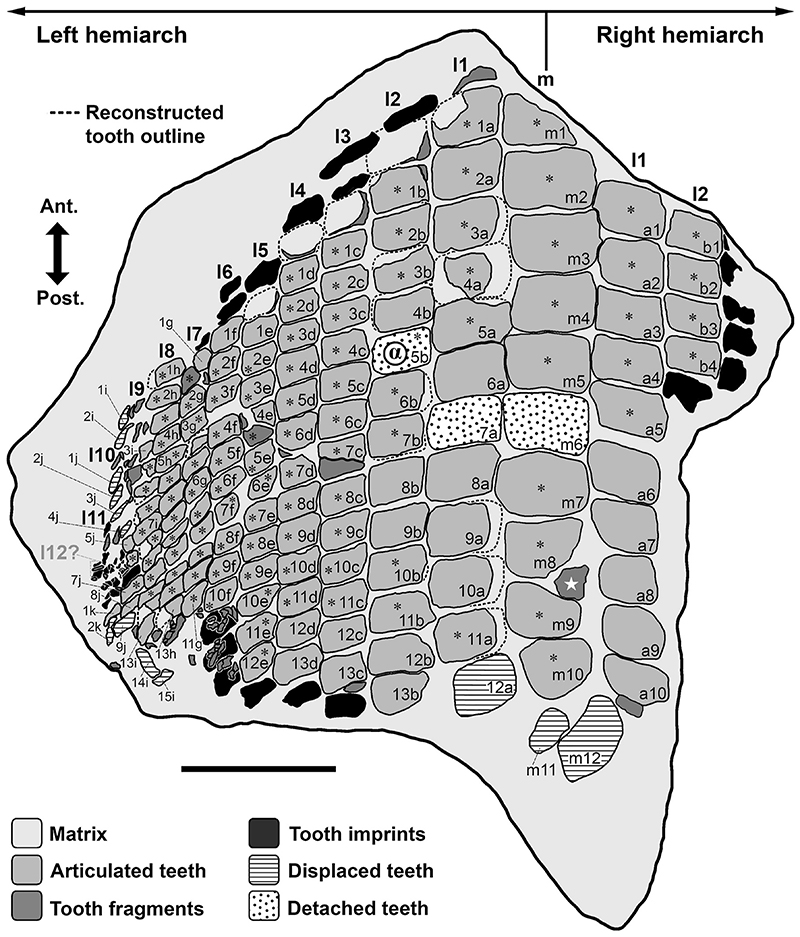
Interpretative line drawing of PIMUZ A/I 5056; the slab is showing several articulated and disarticulated teeth belonging to the lower dentition of †*Ptychodus maghrebianus* sp. nov. Asterisks indicate the worn teeth. A code (e.g. a1, a2, etc.) has been arbitrarily assigned to all the teeth preserved from anterior end of each tooth row. m, symphyseal row; l1-12, lateral rows. α, original placement of the detached tooth “PIMUZ A/I 5056α”. The white star indicate a possible fragment of an upper tooth. Scale bar equals 50 mm.

**Fig. 6:**
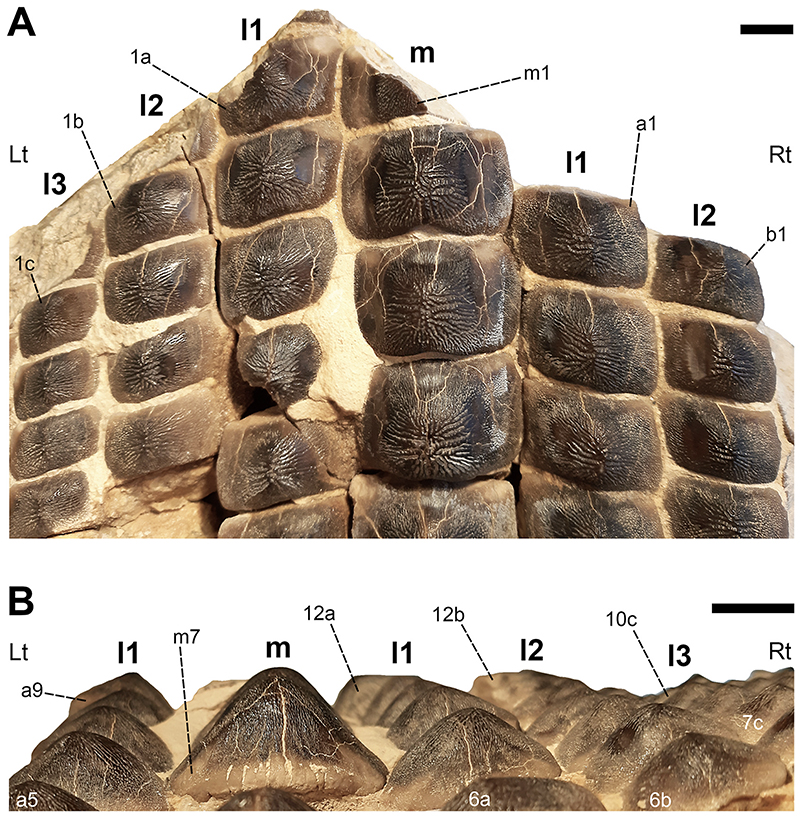
Details of the slab PIMUZ A/I 5056 partially exhibiting the central antero-posterior rows (m, symphyseal; l1-3, lateral) of the lower tooth plate of †*Ptychodus maghrebianus* sp. nov. in occlusal (A) and anterior (B) views. For the tooth codes (e.g. 1a, 1b, etc.) see the “Interpretative line drawing” in Fig. 4. Lt, left side; Rt, right side. Scale bar equals 10 mm.

**Fig. 7:**
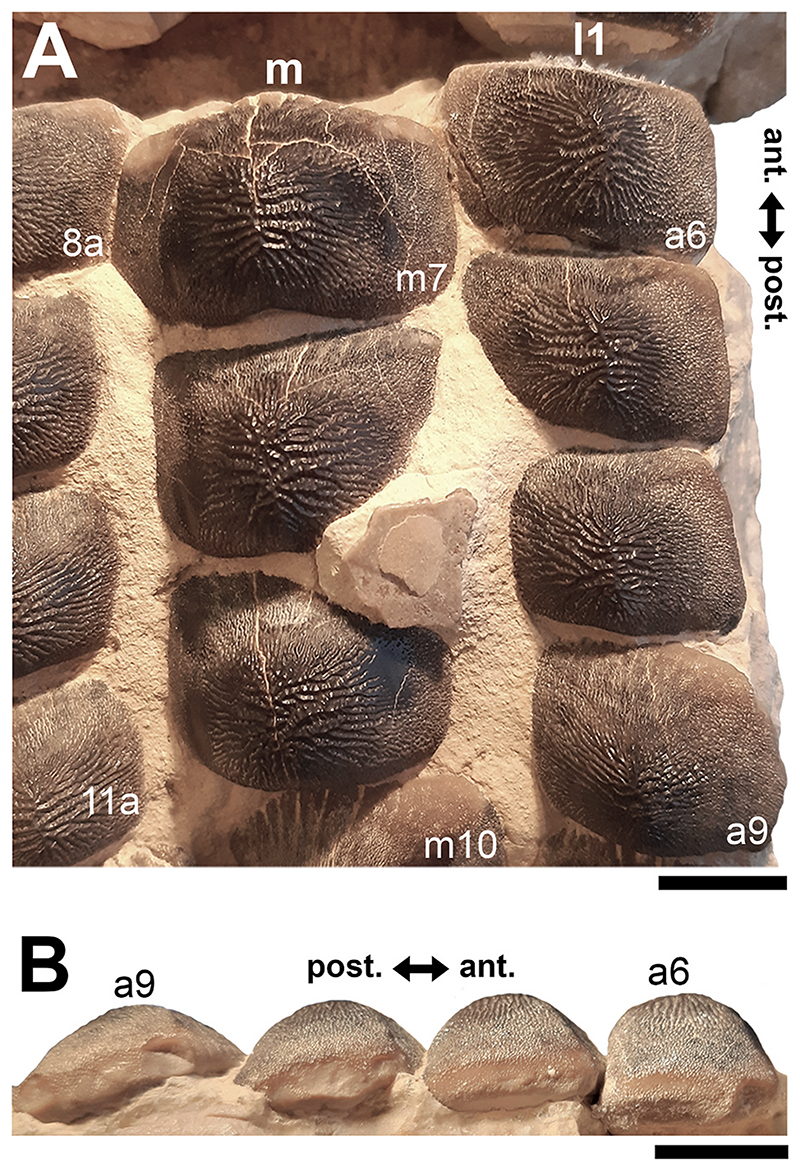
Details of the slab PIMUZ A/I 5056 exhibiting the posterior portion of the tooth rows “m” (symphyseal) and “l1” (lateral, right side) of the lower tooth plate of †*Ptychodus maghrebianus* sp. nov. in occlusal (A) and lateral (B) views. For the tooth codes (e.g. 8a, 11a, etc.) see the “Interpretative line drawing” in Fig. 4. Scale bar equals 10 mm.

**Fig. 8:**
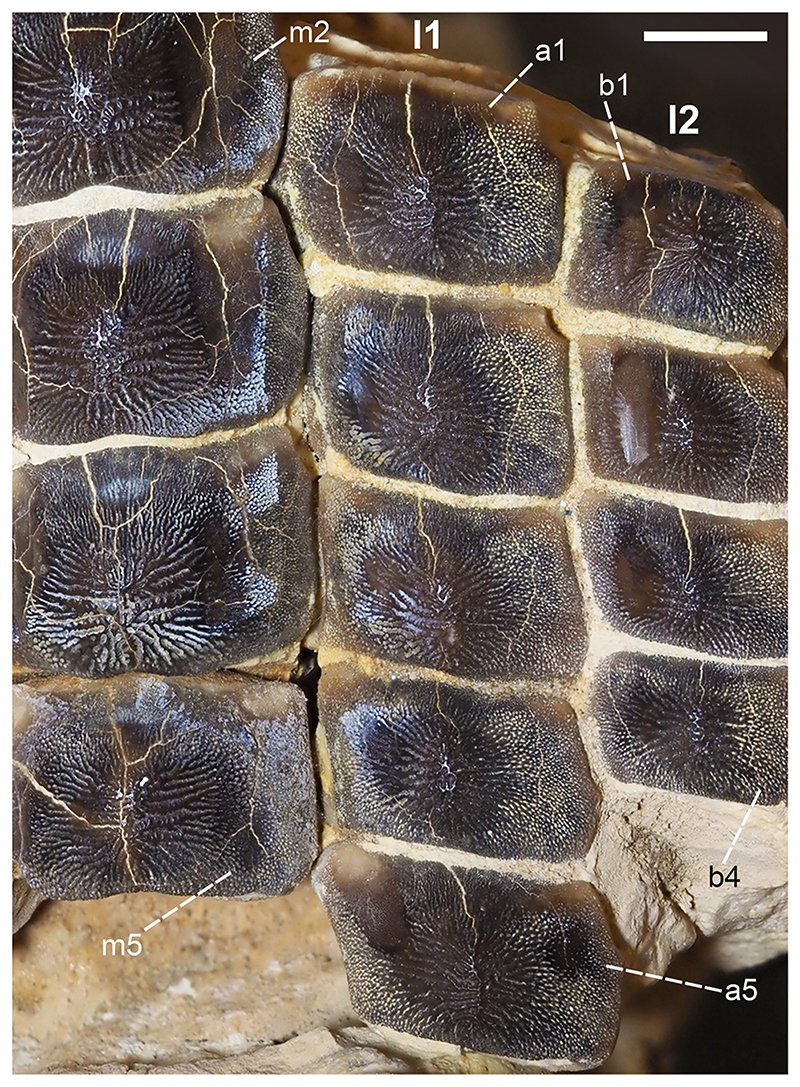
Detail of the slab PIMUZ A/I 5056 exhibiting the anterior portion of the tooth rows “m” (symphyseal) and “l1-l2” (lateral, right side) of the lower tooth plate of †*Ptychodus maghrebianus* sp. nov. in occlusal view. For the tooth codes (e.g. m2, m5, etc.) see the “Interpretative line drawing” in Fig. 4. Scale bar equals 10 mm.

**Fig. 9:**
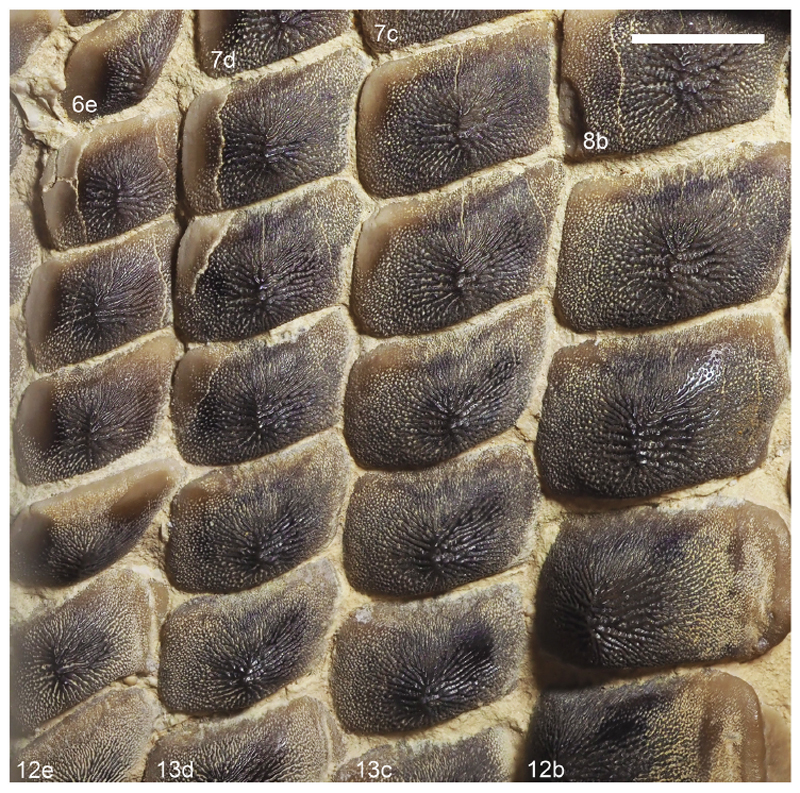
Detail of the slab PIMUZ A/I 5056 exhibiting the latero-posterior portion (left side) of the lower tooth plate of †*Ptychodus maghrebianus* sp. nov. in occlusal view. For the tooth codes (e.g. 6e, 12e, etc.) see the “Interpretative line drawing” in Fig. 4. Scale bar equals 10 mm.

**Fig. 10:**
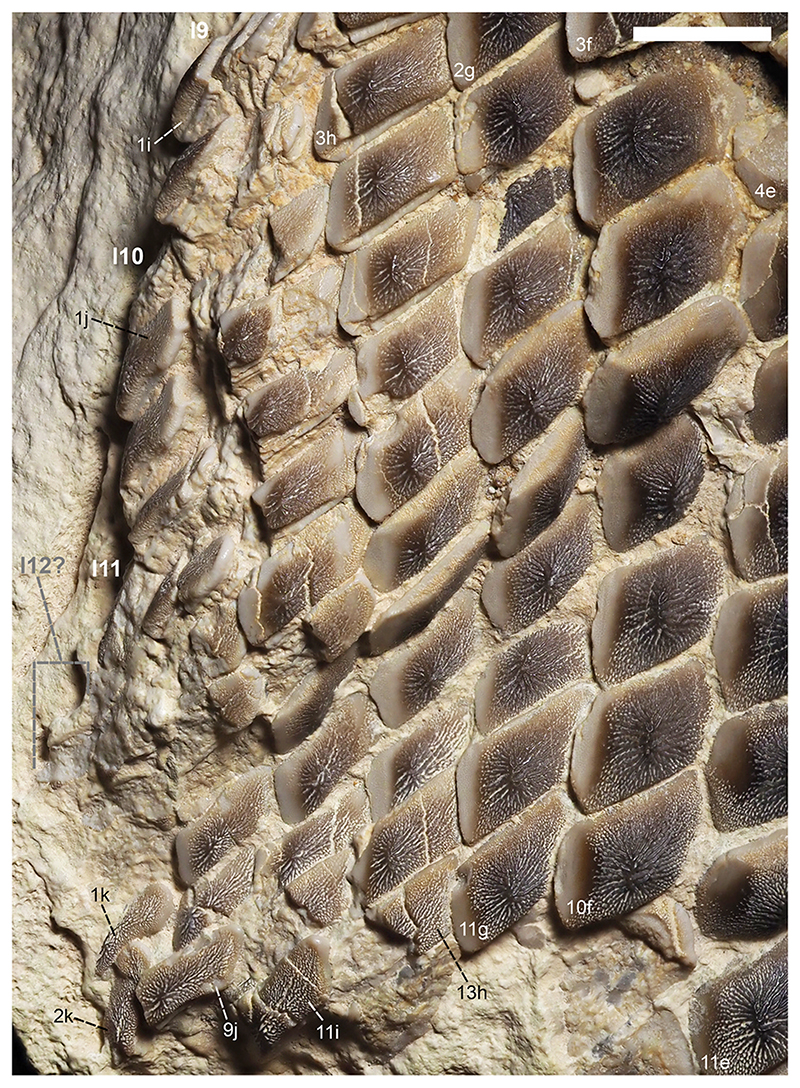
Detail of the slab PIMUZ A/I 5056 exhibiting the distalmost portion (e. g. rows “l8-l11”, left side) of the lower tooth plate of †*Ptychodus maghrebianus* sp. nov. in occlusal view. Fragmentary tooth remains and imprints possibly belonging to the row “l12” are also recognizable. For the tooth codes (e.g. 1h, 1g, etc.) see the “Interpretative line drawing” in Fig. 4. Scale bar equals 10 mm.

**Fig. 11:**
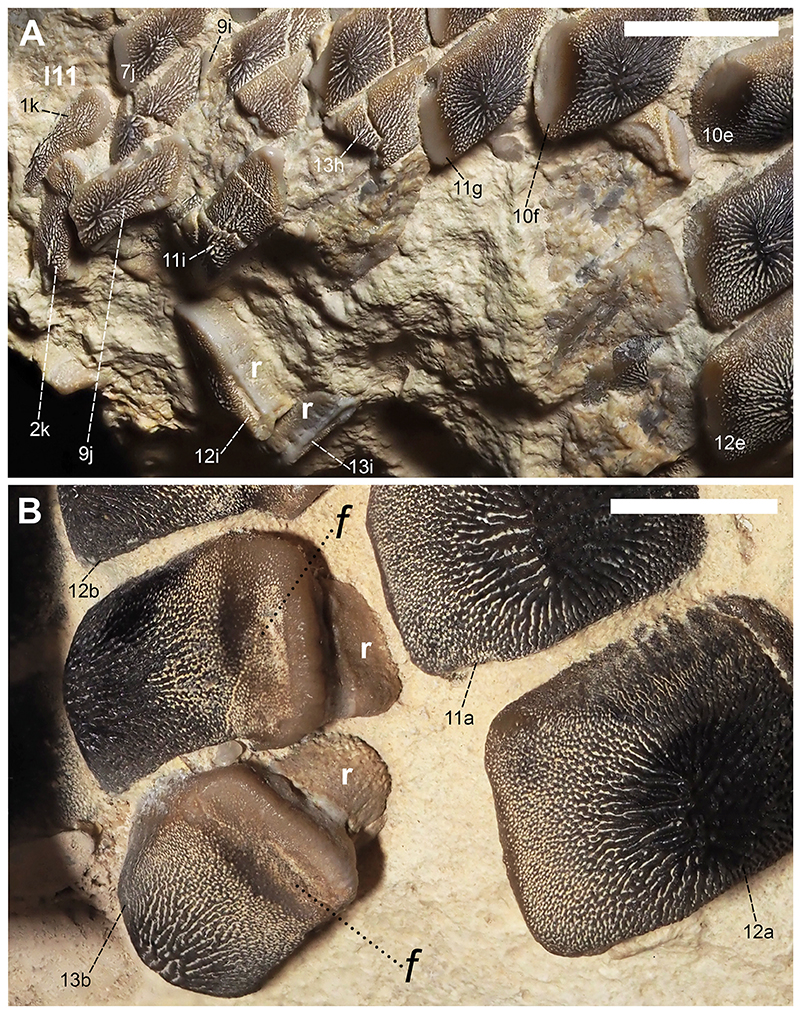
Details of the slab PIMUZ A/I 5056 exhibiting two posterior areas of the lower tooth plate of †*Ptychodus maghrebianus* sp. nov. in occlusal view. A) Distalmost rows (e.g. “l10”, “l11”, left side). B) Central antero-posterior rows. For the tooth codes (e.g. 8i, 11h, etc.) see the “Interpretative line drawing” in Fig. 4 *f*, articular facets; r, tooth roots. Scale bar equals 10 mm.

**Fig. 12:**
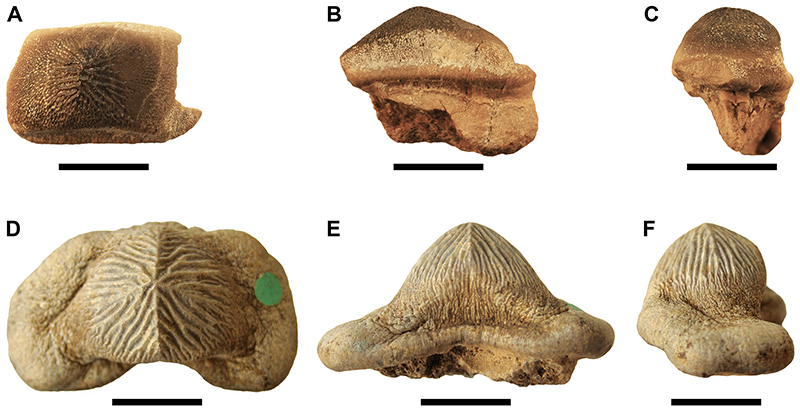
Detached tooth “PIMUZ A/I 5056α” of †*Ptychodus maghrebianus* sp. nov. (A-C) originally articulated within the dentition PIMUZ A/I 5056 (see also “α” in Fig. 4) and holotype (NHMUK OR 28394) of *P. mortoni* Mantell (1836) (D-F). A, D, occlusal views; B, E, posterior views; C, F, lateral views. Scale bar equals 10 mm.

Specimen PIMUZ A/I 5056 (Figs. 4–12) consists of about 140 teeth, most of them still in their original position, being articulated to each other forming 14 antero-posteriorly directed, elongated tooth rows. The lateral margins of the rows are imbricated to each other, composing a large portion of a single dental plate, which is embedded in a massive calcareous nodule. Slight variations of the crown outline occur across the dental plate, while the specie-specific pattern of ornamentation is the same in all preserved teeth (see ‘Diagnosis’, above). The external edges of this dental plate are poorly preserved, and traces, imprints or fragments of other teeth are undoubtedly recognizable all around it indicating that the tooth plate originally was larger (e.g. Figs. 4 and 5). Additionally, three teeth originally arranged within the central row of this plate became detached; two of them must be considered currently lost (see Figs. 4 and 5). The only remaining tooth ‘PIMUZ A/I 5056α’ (Fig. 12) has a polygonal and slightly asymmetric outline consistent with the depression ‘α’ in the dental plate, depicted in Fig. 5. Although the right margin of this tooth is largely damaged, the posterior portion of a lowered, lateral surface is still recognizable (Fig. 12A and B). Similar lowered grooves also are easily detectable on the intact right side of other articulated, or slightly displaced, teeth that are present in the posterior portion of the dental plate (see *f* in Fig. 11B). Few small, parallelogram-shaped teeth are displaced at the posterior portion of the dental plate (on the bottom left in Fig. 10); two of them also exhibit a massive root (see Fig. 11A). Most of the teeth, including ‘PIMUZ A/I 5056α’ (Fig. 12), also display different degrees of wear on their occlusal surfaces (see Figs. 6–10). In general, the most extensive and marked abrasions are on the lateral marginal areas (e.g. Fig. 10) while the largest teeth that are centrally placed within the tooth plate also exhibit slightly worn cusps (Figs. 6–8). The small, parallelogram-shaped teeth arranged in the external rows on the left side in Fig. 10 lack cusps and exhibit only the distal edges, which are completely abraded (see also Fig. 11A). Tooth PIMUZ A/I 5056α also exhibits a very massive root that is damaged on the left side. However, a shallow antero-posteriorly directed sulcus that separates two root lobes is still observable (Fig. 12B). In lateral view (Fig. 12C), the cusp apices are well developed and rounded. The labial face of the tooth roots is inclined posteriorly, while the lingual face is almost perpendicular to the dental crown. For measurements, see Table 2 (see also ‘Data Archiving Statement’, below).

*Remarks*. Despite some minor taphonomic disarticulations (e.g. Fig. 11), PIMUZ A/I 5056 consists of several articulated teeth in their original position belonging to a unique dentition (see Figs. 4 and 5). The largest teeth forming the medial row (m) in Figs. 6 and 7A represent undoubtedly symphyseals of the lower dental plate. Additional 11 rows (l1-l11) still are arranged laterally to the symphyseal row, forming a large portion of the left jaw hemiarch; only two lateral rows belong to the right jaw hemiarch (see Fig. 6). Various tooth fragments and imprints suggest the presence of additional rows originally positioned both antero-posteriorly and mesio-distally within the lower dentition (e.g. Fig. 6A). In particular, tooth imprints belonging to a possible distal row (l12? In Fig. 10) are recognizable on the left, external side of the dental plate. Additionally, a tooth fragment wedged between two lower symphyseal teeth (m8, m9 in Fig. 7A) of the posteriormost region of the dental plate possibly represents the only remain of the upper dentition. This fragment consists of a small portion of a tooth cusp with the occlusal surface placed in the opposite direction compared to those of the articulated lower symphyseals. Roots of most preserved teeth in PIMUZ A/I 5056 are concealed within the matrix. Only a few of them are exposed and uncovered. The isolated tooth PIMUZ A/I 5056α (Fig. 12), which originally was positioned within row l2 (see also ‘α’ in Fig. 5) is about 17 mm in total height, while its root measures about 19 mm in width, 10 mm in length and 8 mm in height. The root height therefore represents about 47% of the total tooth height. Lesser heights were measured for both roots and crowns of the posterior teeth (e.g. 12b and 13b in Fig. 11B) arranged in the same row from which the isolated tooth derived (l2; see Fig. 5). For example, the root of 13b (see Fig. 11B) is about 5 mm in height, which is about 38% of the total height (about 13 mm) of this tooth. There are minimal variations in width and length of the roots along the rows. Measurements of the roots of the posterior teeth belonging to distalmost rows (e.g. l9; see Fig. 5), as well as their overall size are even smaller. For instance, the root of 14i (see Fig. 11A) measures about 11 mm in width and 2.20 mm in height; the root height in this case is about 49% of the total tooth height (4.50 mm).

## 6 Morphological traits

### 6.1 General features

Only a slight heterodonty characterized by weak mesio-distal variations is recognizable in the general crown shape in the dentition of †*Ptychodus maghrebianus* sp. nov. (see Figs. 6– 11). Conversely, the teeth exhibit a quite substantial morphological uniformity along each row. As already was reported for other species (e.g. Hamm, 2017; Amadori et al., 2020a, b), lower symphyseal teeth also represent the most diagnostic dental elements in †*P. maghrebianus* sp. nov. In †*P. maghrebianus* sp. nov., the lower symphyseals have a rectangular crown with a rounded cusp apex. The cusp exhibits irregular and fine ridges that radiate from the central area of the tooth towards the margins and subsequently divide marginally into finer ridges. The radial ridge ramifications increasingly become thinner to finally form a fine granular pattern on the marginal areas. However, diagnostic patterns are not recognizable within the fine marginal ornamentation pattern. Moving mesio-distally and antero-posteriorly along the dental plate, these occlusal ornamentations occur in all teeth without any detectable variations. Both anterior pro-tuberance and posterior sulcus are poorly developed. The cuspidate crown overhangs an anaulacorhizous, bilobate root on all sides. Variations of tooth morphologies between the various rows and arrangement patterns within the lower dental plate of †*P. maghrebianus* sp. nov. are clear (Fig. 4), while no direct evidence of the opposite dentition is preserved.

### 6.2 Lower dentition

The lower dental plate of †*Ptychodus maghrebianus* sp. nov. originally consisted of at least 23 antero-posteriorly directed and parallel rows of teeth, arranged in eleven laterals rows (l1-11) on both sides of the distinct symphyseal row (m; see interpretative illustrations in Fig. 5). No relevant variations in shape, size and occlusal ornamentation are recognizable within each tooth row, except for a gradual increase of the cusp height anteriorly (e.g. Fig. 7B).

Specimen PIMUZ A/I 5056 (Fig. 4) preserves twelve symphyseal teeth having large, rectangular crowns with raised and rounded cusp apices; one of the teeth, however, is lost. The anteriormost symphyseal tooth (m1) is broken (see Fig. 6A), while the last two teeth (m11-12) of this row are still partially covered by matrix (see Fig. 4). The crested apex of lateral teeth (l1-9) continuously is displaced distally and decreases progressively towards the external sides of the dental plate, almost disappearing in the distalmost rows (l10-11). The first pair of lateral rows (l1) consists of transversally elongated and parallelogram-shaped teeth (Fig. 6A). Both anterior protuberance and posterior sulcus are poorly developed (Fig. 7A). Moreover, their mesial edge is depressed to allow the imbrication with the symphyseal teeth (see Fig. 8). The dental root is massive and bilobate with the lateral side being folded distally and the labial face posteriorly inclined. The general shape and the ‘articular facets’ described for the teeth belonging to row l1 are maintained in the subsequent rows (l2-11), while the thickness of the crown, the height of the cusps and the tooth size decrease mesiodistally (see also ‘Data Archiving Statement’, below). Rows l2-9 (Figs. 7–10) exhibit tooth crowns with low and faintly pointed cusps. The distalmost teeth (rows l10-11) do not have a developed cusp and are the most transversally elongated of all teeth (Fig. 10). The roots of teeth belonging to the distalmost rows (e.g. l9) miss the antero-posterior sulcus, but remain very massive (see Fig. 11A). These lateral teeth also have a reduced anterior protuberance only presenting a slightly convex margin, while their posterior sulcus is shallow (Figs. 10 and 11A). A summary of tooth measurements for each lower row is provided in Table 2.

## 7 Statistical analyses

### 7.1 Mesiodistal tooth patterns

**Fig. 13:**
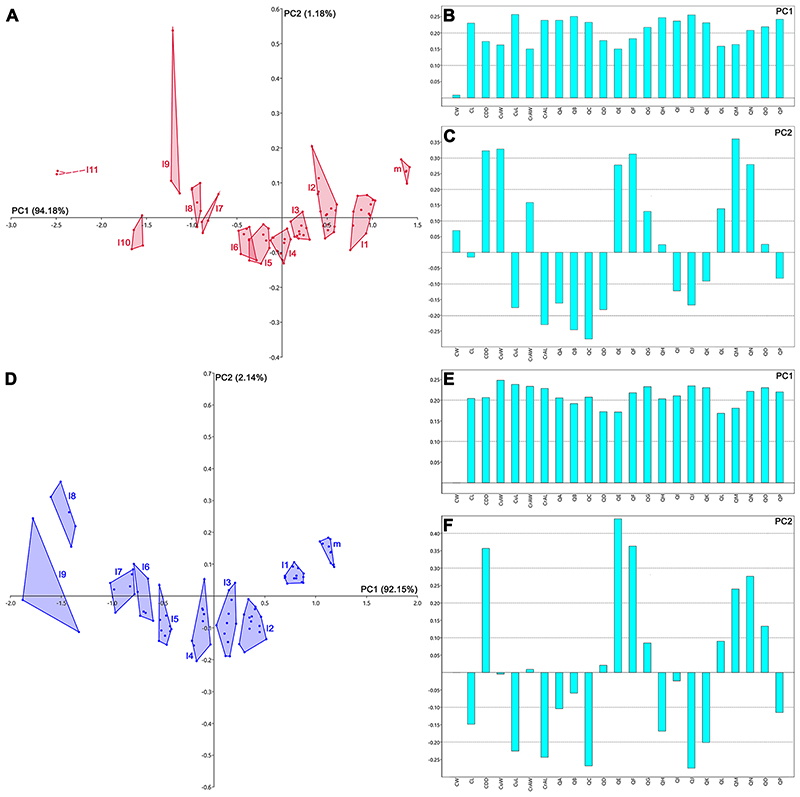
Results of PCA performed on 23 linear measurements from and represented as convex hulls. Morphospaces related to the intraspecific variability (mesiodistal pattern) of †*Ptychodus maghrebianus* sp. nov. (A) and *P. mortoni* Mantell (1836) (D). Loading values (B, C, E, F) showing the variables associated with the first two PC axes.

The PCA performed on 91 articulated teeth belonging to the lower dentition of †*Ptychodus maghrebianus* sp. nov. described here (PIMUZ A/I 5056, see Fig. 4) detected 23 PCs, with only the first explaining more than 5% of variation and accounting for 94.2% of the total variability (see Appendix Multimedia component 1, Supplementary online material). PC1 (94.2%) is mainly related to CL, CuL, CrAL, QA-QC, QG-QK and QN-QP (for acronyms, see Fig. 3), with positive values related to the teeth belonging to rows m, l1-l3 and, partially, l4. Teeth of rows l5-l11 conversely are distributed along negative values (see Fig. 13A–C). PC2 (1.2%) is related to CDD, CuW, QE, QF, QM and QN (for acronyms, see Fig. 3). Positive scores of PC2 are related to both symphyseal and lateral teeth belonging to the rows m, l1, l2, l8, l9, l11 (see Fig. 13A). See Appendix Multimedia component 2 (Supplementary online material) for the results of nonparametric tests (PERMANOVA and ANOSIM).

Specimen ROM 21705 (see Shimada, 2012, Fig. 4) consists of more than 200 articulated teeth and represents the most complete lower dentition of †*Ptychodus mortoni* so far described. The PCA performed on 90 selected teeth from this exceptional find (ROM 21705) identified 23 PCs. Among them, only the first represent more than 5% of the variation, accounting for 92.3% of the total variability, similar to the condition seen in †*P. maghrebianus* sp. nov. (see Appendix Multimedia component 3, Supplementary online material). PC1 (92.3%) is mainly related to CL, CuL, CrAL, QA, QC, QF-QK and QN-QP (for acronyms, see Fig. 3) as in †*P. maghrebianus* sp. nov., but additionally also to CDD, CuW, CrAW (for acronyms, see Fig. 3G). The teeth from rows m, l1-l3 are arranged along the positive values of PC1, while negative values are related to teeth of rows l4-l11 (see Fig. 13D–F). PC2 (2.1%) is related to CDD, QE and QF (for acronyms, see Fig. 3) as also in †*P. maghrebianus* sp. nov. and both medial and distal teeth (rows m, l1, l7, l8) are distributed along its positive scores. See Appendix Multimedia component 4 (Supplementary online material) for the results of nonparametric tests (PERMANOVA and ANOSIM).

**Fig. 14:**
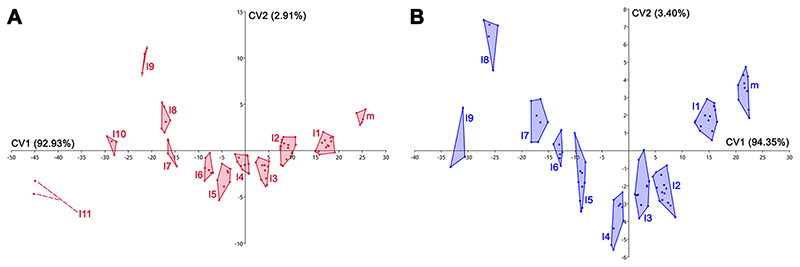
Results of CVA performed on 23 linear measurements and represented as convex hulls. Morphospaces related to the intraspecific variability (mesio-distal pattern) of †*Ptychodus maghrebianus* sp. nov. (A) and †*P. mortoni* Mantell (1836) (B).

The CVA analysis performed on the same samples used for the PCAs (see above) confirms the mesiodistal pattern with clearly separated morphospaces distributed along the CV1 scores for each antero-posterior row of both †*Ptychodus maghrebianus* sp. nov (m, l1-l11; see Fig. 14A). and †*P. mortoni* (m, l1-l9; see Fig. 14B). See Appendixes Multimedia component 5 and 6 (Supplementary online material) for significant comparisons (p < 0.05; Hotelling’s *t*-test).

### 7.2 †*Ptychodus maghrebianus sp. nov. vs* †*P. mortoni Mantell (1836)*

**Fig. 15:**
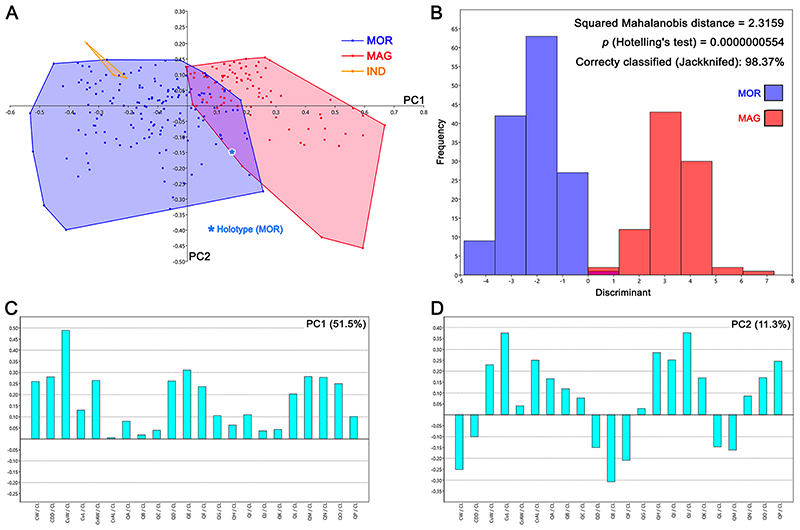
Results of PCA (A, C, D) and LDA (B) related to the interspecific variability between †*Ptychodus maghrebianus* sp. nov. (MAG) and †*P. mortoni* Mantell (1836) (MOR) and represented as convex hulls. Morphospaces plotted on PC1 and PC2 and based on the entire measurement sample; indeterminate teeth (IND) are also shown. Loading values (C, D) showing the variables associated with the first two PC axes.

**Fig. 16:**
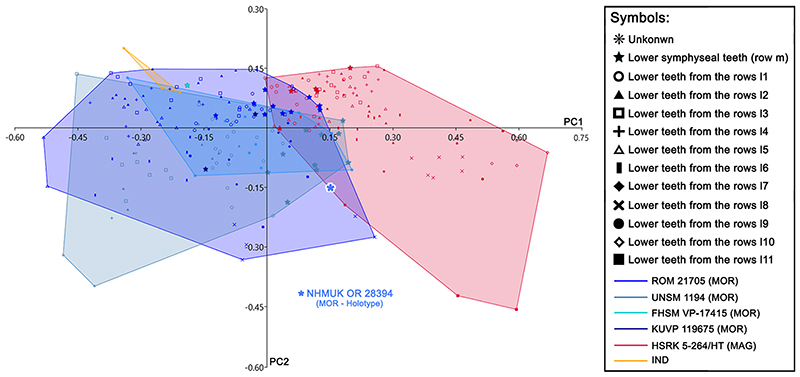
Results of PCA related to the interspecific variability between six articulated or associated dentitions of †*Ptychodus maghrebianus* sp. nov. (PIMUZ A/I 5056), †*P. mortoni* (FHSM VP-335, FHSM VP-17415, KUVP 119675, ROM 21705 and UNSM 1194) and three isolated teeth previously assigned to †*Ptychodus* sp. (IND) and represented as convex hulls. Morphospaces of the single specimens are plotted on PC1 and PC2 and based on 23 linear measurements. For the loading values see Fig. 15C and D.

The principal component analyses (PCA, see Fig. 15A; see also Appendix Multimedia component 9, Supplementary online material) performed on the pairwise comparison between †*Ptychodus maghrebianus* sp. nov. and †*P. mortoni* detected 22 PCs, with only the first four explaining more than 5% of variation and accounting together for 75.5% of the total variability (see Appendix Multimedia component 7, Supplementary online material). The analysed samples comprise 248 teeth and belong to the articulated holotype of †*P. maghrebianus* sp. nov. (PIMUZ A/I 5056), to four articulated/associated specimens of †*P. mortoni* (FHSM VP-335, FHSM VP-17415, KUVP 119675, ROM 21705 and UNSM 1194) and to three isolated teeth previously assigned to †*Ptychodus* sp. (see Table 1 for more details). PC1 (51.5%) is mainly related to the relationship between the width of the cusp (CuW) and the antero-posterior length of the entire crown (CL; see Fig. 15C). The positive values of PC1 are confined to the teeth of †*P. maghrebianus* sp. nov., while teeth of †*P. mortoni* lie along negative values (see Fig. 15A; see also Appendix Multimedia component 9, Supplementary online material). PC2 (11.3%) is mainly related to the relationship between the length of the cusp (CuL) and the length of the crown (CL), as well as to the relationship between one of the measurements describing the posterior outline of the crown (QJ in Fig. 3G) and the length of the crown (CL; see Fig. 15D). PC3 (6.8%) is mainly related to the relationship between one of the measurements describing the antero-mesial outline of the crown (QD in Fig. 3G) and the length of the crown (CL; see Appendix Multimedia component 9, Supplementary online material). PC4 (5.4%) is mainly related to the relationship between three of the measures describing the anterior and the posterior outline of the crown (QB, QH and QP in Fig. 3G) and the length of the crown (CL; see Appendix Multimedia component 9, Supplementary online material). Both species share almost the same range of values along PC2, PC3 and PC4 with only few exceptions (see Fig. 15A; see also Appendix Multimedia component 9, Supplementary online material). Accordingly, †*Ptychodus maghrebianus* sp. nov. is almost completely confined in positive PC1 scores,whereas †*P. mortoni* mostly lies along negative PC1 scores. The overlap of their morphospaces is related to PC1 values between – 0.1 and 0.3. The three indeterminate teeth are mainly distributed within the morphospace of *†Ptychodus mortoni* and along the PC1 values between – 0.2 and — 0.4 (see Fig. 15A; see also Appendix Multimedia component 9, Supplementary online material). The LDA correctly assigned more than 98% of teeth to their a priori assumed groups (see Fig. 15B). The morphospace distribution of each specimen (FHSM VP-335, FHSM VP-17415, KUVP 119675, NHMUK OR 28394; ROM 21705 and UNSM 1194) assigned to †*P. mortoni* is largely overlapping (see PCA in Fig. 16; see also Appendix Multimedia component 10, Supplementary online material). The distribution of the teeth from different antero-posterior rows within each morphospace exhibits a slightly larger overlap for teeth from the central portion (e.g. rows m and l1) of the lower dental plates (see Fig. 16; see also Appendix Multimedia component 10, Supplementary online material). See Table 3 for the results of nonparametric tests (PERMANOVA and ANOSIM).

**Fig. 17:**
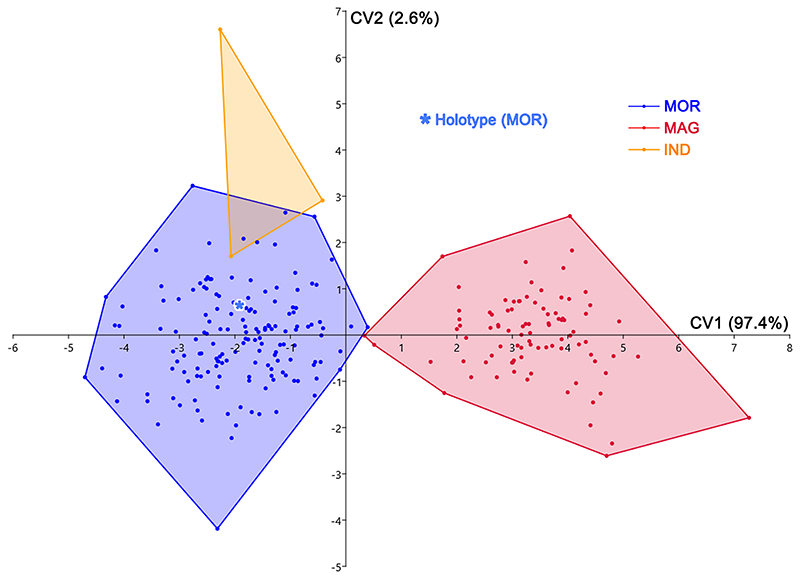
Results of CVA related to the interspecific variability between *Ptychodus maghrebianus* sp. nov. MAG) and †*P. mortoni* Mantell (1836) (MOR), based on 23 linear measurements and represented as convex hulls; indeterminate teeth (IND) are also shown.

The overlap of morphospaces between the two species (†*Ptychodus maghrebianus* sp. nov. and †*P. mortoni*) is instead completely absent in the CVA performed on the same tooth sample (see Fig. 17). †*Ptychodus* *mortoni* mostly lies along negative CV1 (97.4%) scores, while †*P. maghrebianus* sp. nov. is exclusively limited to positive values. Both taxa cover approximately the same score range along CV2 (2.6%). The isolated specimen NHMUK OR 28394 (holotype) largely falls within the negative range of CV1 occupied by †*P. mortoni* (see Fig. 17). The indeterminate teeth (MT-G-011, MT-G-012 and MT-G-013) also are clearly distributed along the negative CV1 values. Significant comparisons (p < 0.05, Hotelling’s *t*-test) are reported in Table 4.

**Fig. 18:**
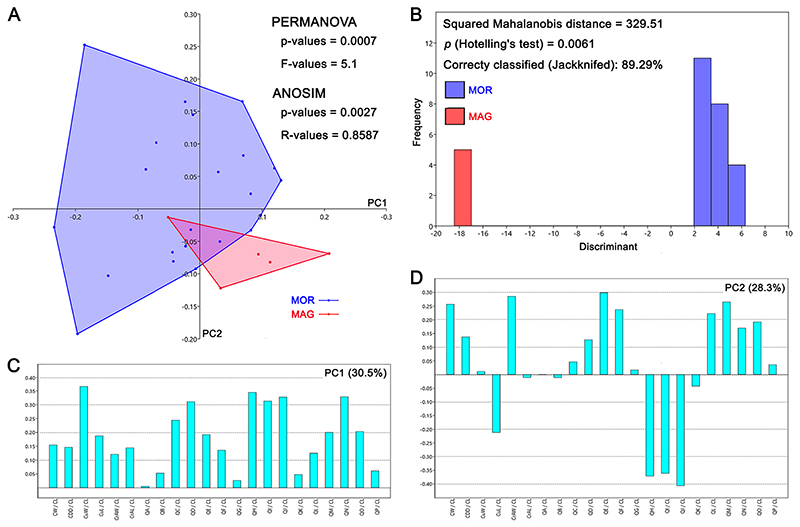
Results of PCA (A) and LDA (B) related to the interspecific variability between lower symphyseal teeth of †*Ptychodus maghrebianus* sp. nov. (MAG) and *P. mortoni* Mantell (1836) (MOR) and represented as convex hulls. Morphospaces plotted on PC1 and PC2 and based on symphyseal measurement sample. Loading values (C, D) showing the variables associated with the first two PC axes.

The PCA (Fig. 18A; see also Appendix Multimedia component 11, Supplementary online material) performed on the symphyseal teeth of †*Ptychodus maghrebianus* sp. nov. and †*P. mortoni* detected 22 PCs. Among them, the first four explain more than 5% of the variation and account together for 82.9% of the total variability (see Appendix Multimedia component 8, Supplementary online material). The analysed sample consists of 28 symphyseal teeth coming from five articulated/associated specimens (PIMUZ A/I 5056, FHSM VP-17415, KUVP 119675, ROM 21705 and UNSM 1194; see Table 1 for more details). PC1 (30.5%) is mainly related to the relationship between the width of the cusp (CuW) and the antero-posterior length of the entire crown (CL), as well as to the relationship between the measurements describing the posterior outline of the crown (QH in Fig. 3G) and the length of the crown (CL; see Fig. 18C). PC2 (28.3%) is mainly related to the width of the crown (CW), the width of the crested area (CrAW) and two of the measurements describing both the mesial and distal outlines of the crown (QE and QM in Fig. 3G) and their relationship to the length of the crown (CL; see Fig. 18D). Teeth of †*P. maghrebianus* sp. nov. mostly lie along positive values of PC1 and negative values of PC2, while the morphospace of †*P. mortoni* ranges from negative to positive values of both PCs. PC3 (13.8%) is mainly related to the relationship between the width of the crested area (CrAW) and the length of the crown (CL; see Appendix Multimedia component 11, Supplementary online material). †*Ptychodus maghrebianus* sp. nov. covers significantly lower values than †*P. mortoni* along PC3 with morphospaces never overlapping each other. PC4 (10.4%) is mainly related to the relationship between the length of the crested area (CrAL) and the length of the crown (CL; see Appendix Multimedia component 11, Supplementary online material). The range of †*P. maghrebianus* sp. nov. values is largely contained by the distribution of †*P. mortoni* along PC4 (see Appendix Multimedia component 11; Supplementary online material). Nevertheless, the LDA correctly assigned more than 89% of teeth to their a priori suggested groups (see Fig. 18B).

## 8 Discussion

### 8.1 Comparative Remarks

#### 8.1.1 Traditional identification approach

†*Ptychodus mortoni* Mantell (1836) (holotype in Fig. 12D-F) is the most morphologically similar taxon to †*P. maghrebianus* sp. nov. based on the shared radial patterns of their occlusal ridges. Teeth of †*P. mortoni* have a conical and sharp-tipped cusp, which is conversely rounded and blunt in †*P. maghrebianus* sp. nov. (see Fig. 12). Additionally, in †*P. mortoni*, the cusp is limited to the central part of the tooth crown and is flanked on both sides by two depressed and flat lateral areas that often are curved downwards (see Fig. 12). The occlusal surface of teeth of †*P. maghrebianus* sp. nov., conversely, is almost totally occupied by the cusp except for the mesial articular facet in lateral teeth (e.g. Figs. 6B and 12B). The posterior sulcus is deeper and more marked in †*P. mortoni* than in †*P. maghrebianus* sp. nov. Moreover, the lateral parallelogram-shaped teeth of †*P. maghrebianus* sp. nov. markedly differ in occlusal outline from those of †*P. mortoni*, which are distally curved. In †*P. mortoni*, the most distal teeth of the dentition exhibit small occlusal cusps, while they have almost flat crowns in *P. maghrebianus* sp. nov. Additionally, these two taxa exhibit different transitions between ridges and marginal ornamentations. The radial ridges on the tooth crown of †*P. mortoni* are mostly limited to the cusp and are readily distinguishable from the marginal ornamentation, whereas they gradually merge with the lateral granulation in †*P. maghrebianus* sp. nov. Diverse arrangements characterize the lower dentition of the two species with the number of lateral antero-posterior rows in †*P. maghrebianus* sp. nov. being higher (11) than in *P*. *mortoni* (9). Furthermore, †*P. maghrebianus* sp. nov. exhibits a narrower symphyseal tooth row in relation to the total width of the lower dental plate (see also “Reconstruction of the lower dental plate”, below).

The isolated teeth assigned to †*Ptychodus* sp. by Carrillo (2009) differ markedly from those of †*P. maghrebianus* sp. nov. by exhibiting a very high, conical and pointed cusp. Carrillo (2009) described a greater sharpening of the cusps of these isolated teeth compared to that observed in †*P. mortoni*, hypothesizing that they belong to a new species. Their morphological proximity is obvious, but both differ in the morphology of the cusps. However, the description of excellent comparative material (e.g. articulated dentitions of †*P. mortoni*) and the traditional investigations performed up to now have been insufficient to properly identify these isolated specimens (see also Shimada, 2012).

#### 8.1.2 Statistical approach

Since their first discoveries in the XVIII^th^ century, the study of †*Ptychodus* teeth has been based on a restricted number of morphological characters (e.g. overall shape and occlusal ornamentations), which are not useable for quantitative analysis. Such analyses therefore are rarely employed when studying teeth of †*Ptychodus* and traditional taxonomic approaches generally are employed (see Woodward, 1912; Cappetta, 2012; Brignon, 2019; Amadori et al., 2020a). PCA analyses for assessing intraspecific variabilities in low-crowned species of †*Ptychodus* and based on limited data from isolated and/or associated tooth sets are the few exceptions up to now (see Hamm, 2010; Amadori et al., 2020b). Hamm (2010) performed a principal component analysis (PCA) based on three-dimensional morphometric measurements taken from 57 teeth belonging to five disarticulated specimens assigned to †*P. marginalis*. Most of these low-crowned teeth group together, showing no separation into different morphospaces (see Hamm, 2010, Fig. 9). Conversely, Amadori et al. (2020b) recognized a clear tooth pattern in the performed PCA based on linear measures of 23 associated teeth of †*P. latissimus*. Indeed, most of the low-crowned teeth of this species separate well into three different morphospaces (s, symphyseal; l, lateral; d, distal) following a mesiodistal pattern (see Amadori et al., 2020b, Fig. 14).

Statistical analyses (PCA and CVA) performed here and based on linear measurements (see ‘Data Archiving Statement’, below) obtained from the still articulated dentitions of †*Ptychodus maghrebianus* sp. nov. (PIMUZ A/I 5056) and †*P. mortoni* (ROM 21705) highlight tooth morphospace patterns along PC1 with mesially arranged teeth confined to the positive scores and the distalmost ones distributed along negative scores (see Figs. 13 and 14; see also ‘Statistical Analyses’, above). In both species, a mesiodistal pattern thus is clearly recognizable. Both PERMANOVA and ANOSIM strongly support the separation of both †*P. maghrebianus* sp. nov. and †*P. mortoni* into different morphospaces (p-value < 0.05; (see Appendix Multimedia components 2 and 4, Supplementary online material). Although the CVAs confirm these results in both taxa (see Fig. 14), the Hotelling’s test failed for most of the pairwise comparisons (see Appendix Multimedia components 5 and 6, Supplementary online material).

The different morphospace occupations of †*P. maghrebianus* sp. nov. and †*P. mortoni* resolved herein (PCAs in Figs. 15 and 16 and CVA in Fig. 17) support their taxonomic separation into two different species. In particular, the ratio between the width of the cusp (CuW) and the antero-posterior length of the entire crown (CL; see Fig. 15A; see also Appendix Multimedia component 9, Supplementary online material) tends to be larger in †*P. maghrebianus* sp. nov. than in †*P. mortoni*. Both PERMANOVA and ANOSIM also clearly support the different morphospace occupations, showing that measurements are useful to separate the two species (p-value < 0.05; see Table 3). No significant differences, conversely, were detected between the indeterminate group and †*P. mortoni* (p-value > 0.05; see Table 3). The LDA in Fig. 15B significantly confirms the separation of †*P. maghrebianus* sp. nov. from †*P. mortoni* (Hotelling’s test: p-value < 0.05) with more than 98% of teeth being correctly assigned to their a priori groups.

The holotype (isolated tooth) of †*P. mortoni*, NHMUK OR 28394, falls into the morphospace of †*P. mortoni* even when the separation between groups is maximized through the CVA (Fig. 17). Although the group of indeterminate teeth is significantly separated from †*P. mortoni* (Hotelling’s test: p-value <0.05; see Table 4), they nevertheless fall within the range of †*P. mortoni* along PC1. Moreover, they are identified as belonging to †*P. mortoni* in the ‘confusion matrix’ for the CVA (93.15% teeth correctly classified; see Table 5).

The associated/articulated tooth sets assigned to †*P. mortoni* (FHSM VP-335, FHSM VP-17415, KUVP 119675, NHMUK OR 28394; ROM 21705 and UNSM 1194) largely share the same morphospace. Their morphospace overlap with †*P. maghrebianus* sp. nov. is reduced to a very small area mostly including symphyseal teeth of both species (see Figs. 15 and 16; see also Appendix Multimedia components 9 and 10, Supplementary online material). Nevertheless, a clear separation between †*P. maghrebianus* sp. nov. and †*P. mortoni* is still evident if the study sample is limited to the symphyseal teeth (see Fig. 18; see also Appendix Multimedia component 11, Supplementary online material), which is strongly supported by both PERMANOVA and ANOSIM (p-value < 0.05). These results are significantly supported by LDA (Hotelling’s test: p-value < 0.05; see Fig. 18A). Furthermore, around 89% of teeth are correctly assigned to †*P. maghrebianus* sp. nov. and †*P. mortoni* (a priori groups) through the LDA based on symphyseal data only.

### 8.2 Reconstruction of the lower dental plate

Woodward (1904) and Shimada et al. (2009) documented lower dentitions of both low-crowned and high-crowned †*Ptychodus* species that still are articulated with the Meckel’s cartilages. According to these articulated findings, the lower dentition of †*Ptychodus* exhibits a symphyseal row composed of the largest teeth within the dental plate, while teeth decrease in size laterally (see also Woodward, 1887, 1912; Williston, 1900a, b; Everhart and Caggiano, 2004; Shimada, 2012; Diedrich, 2013; Amadori et al., 2020a, b; Hamm, 2020). The articulated tooth set PIMUZ A/I 5056 (Fig. 4) described here allows a detailed assessment of the lower dental plate of †*P. maghrebianus* sp. nov. in its original configuration, although it exhibits slight evidences of post-mortem disturbance (‘disturbed tooth set’, *sensu* Shimada, 2005; see Fig. 5). This significantly contributes to our understanding of the tooth arrangement patterns in these enigmatic sharks.

**Fig. 19:**
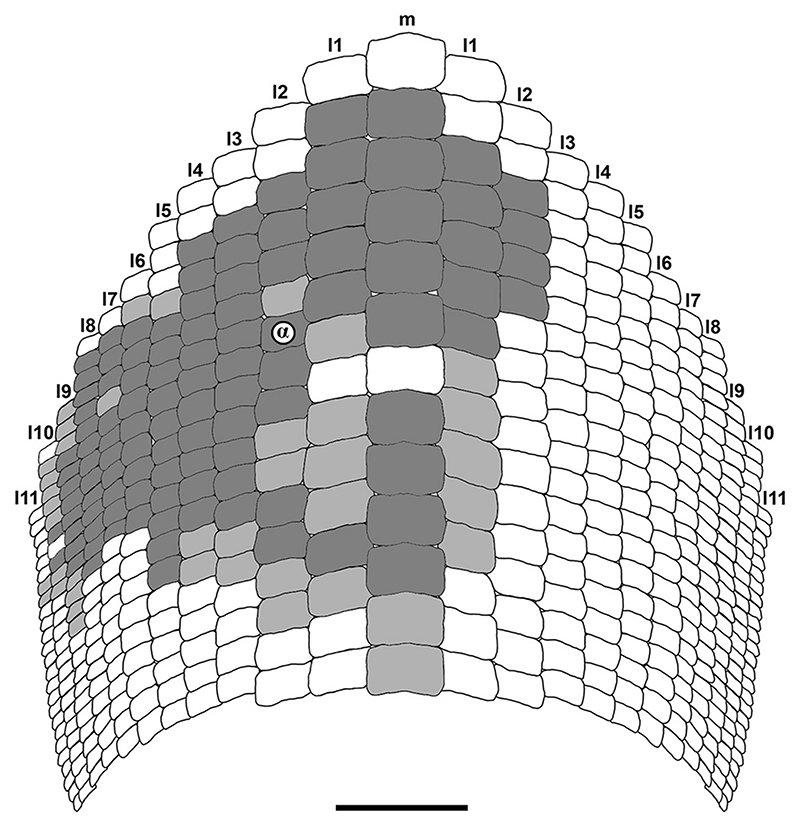
Interpretative reconstruction of the lower tooth plate of †*Ptychodus maghrebianus* sp. nov. based on the articulated teeth (both shades of grey) preserved within the specimen PIMUZ A/I 5056 (holotype). The placement of worn teeth on the articulated specimen is also highlighted (dark grey). m, symphyseal row; l1-l11, lateral rows; α, detached tooth “PIMUZ A/I 5056α”. Scale bar equals 50 mm.

In occlusal view, the tooth plate is semi-elliptic in shape (Fig. 19) and measures ca. 28 cm in width and 30 cm in antero-posterior length. Its anterior and posterior edges seem to be curved with a convex anterior margin similar to that found in other species of †*Ptychodus* (e.g. †*P. decurrens*, †*P. mediterraneus*, †*P. mortoni*; see also Woodward, 1904, 1912; Shimada, 2012; Amadori et al., 2020a *a*). However, the original size and shape of the lower dental plate remain doubtful because of its poorly preserved outer margins (see Fig. 5). The reconstructed dental plate of †*P. maghrebianus* sp. nov. proposed herein (see Fig. 19) originally comprised ca. 465 lower ‘functional teeth’ (*sensu* Shimada, 2012) arranged in a symphyseal row (m) flanked on both sides by 11 lateral rows (l1-11). Based on tooth fragments and imprints, the presence of a possible additional distalmost row (l12) within each hemiarch is hypothesized here (see Figs. 5 and 10). No ‘replacement teeth’ were identified in PIMUZ A/I 5056. Nevertheless, ‘replacement teeth’ represent about 12% of the entire lower dentition in other cuspidate species, such as †*P. mortoni* (see Shimada, 2012). Assuming the same proportion for †*P. maghrebianus* sp. nov., its complete mandibular plate thus would consist of about 521 teeth. The complete dentition of †*P. mortoni* is estimated to include about 250 upper and 300 lower teeth (Shimada, 2012). If an identical ratio (0.83) is applied as ratio to upper and lower teeth of †*P. maghrebianus* sp. nov., its entire dentition would include about 950 teeth. The typical mesio-distal heterodonty (*sensu* Cappetta, 2012) found in †*Ptychodus* mainly consisting of morphological variations of the crown shape, biometric proportions and overall thickness also characterizes the lower dentition of †*P. maghrebianus* sp. nov.

Additionally, various degrees of a distal shift of the occlusal cusp are present in lateral teeth of this cuspidate species. The only putative evidence of the upper dentition is a crown fragment, which is wedged in between two lower symphyseal teeth (m8, m9; see Fig. 7A). This small tooth fragment might represent the rounded, apical portion of a tooth belonging to the first lateral row of the upper dentition. Its placement suggests a tooth occlusion like that proposed for †*P. mortoni* by Shimada (2012) where the cusp apices do not contact during closure of the jaws, but the teeth rather interlock. However, this ‘misaligned occlusion’ cannot be confirmed for †*P. maghrebianus* sp. nov. due to the lack of its upper dentition. The tilted anterior side of the root in lateral teeth of †*P. maghrebianus* sp. nov. (e.g. ‘PIMUZ A/I 5056α’ in Fig. 12C) suggests a rostro-caudal curvature of the lower dental plate. The folded lateral sides also indicate a mesiodistal convexity of this tooth plate, as already hypothesized for other species of †*Ptychodus* (e.g. †*P. mediterraneus*; see Amadori et al., 2020a).

**Fig. 20:**
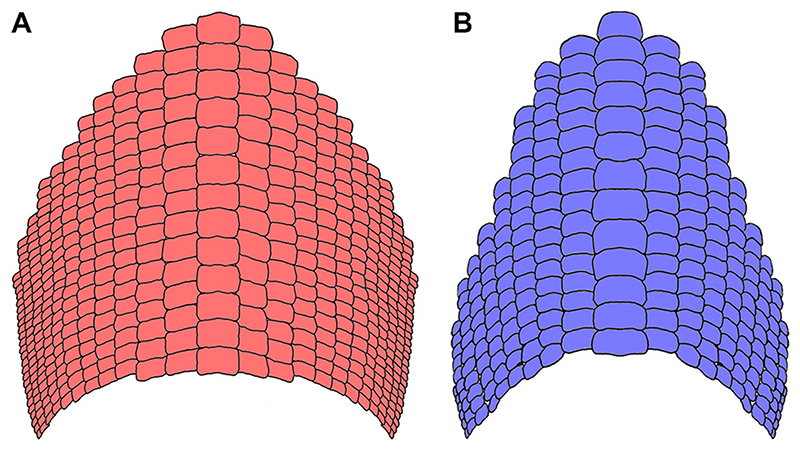
Interpretative reconstruction of the lower tooth plates of †*Ptychodus maghrebianus* sp. nov. (A) and *P. mortoni* Mantell (1836) (B, modified after Shimada, 2012). The “replacement teeth” are not represented in both interpretative drawings.

The proportion between width and length of the dentition of †*Ptychodus*, as well as the number of antero-posterior tooth rows, is variable and probably subject to ontogenetic variations and sexual dimorphism, as already observed in other durophagous elasmobranchs (e.g. *Heterodontus*; Reif, 1976; Powter et al., 2010). Nevertheless, the specimens documented herein (PIMUZ A/I 5056) exhibits a dental plate

composed of the highest number of tooth rows (one symphyseal and 22 laterals) hitherto described for †*Ptychodus*. †*Ptychodus maghrebianus* sp. nov. would therefore have an unusually enlarged dentition with wider distal portions compared to other species (e.g. †*P. mortoni*; see Fig. 20).

### 8.3 Functional specialization

Elasmobranchs exhibit an enormous variability in tooth shapes with distinctive morphological traits, especially among large predatory groups, that also have taxonomic implications (Cappetta, 2012; Corn et al., 2016). For instance, the evolution of ‘pavement-like’ tooth sets suitable for processing hard-shelled prey (durophagy) independently occurred numerous times in their evolutionary history (see Summers et al., 2004; Amadori et al., 2020a). Dentitions of durophagous sharks mainly consist of molariform, and generally relatively flat-crowned, teeth that are closely interlocked (e.g. Woodward, 1912; Summers, 2000; Cappetta, 2012; Kolmann et al., 2015; Amadori et al., 2020a, b; Hamm, 2020). Several lineages of these ‘hard-shelled prey specialists’ evolved and diversified exhibiting extremely variable tooth morphologies and arrangements, widely described in extant and fossil taxa (Woodward, 1912; Wilga and Motta, 2000; Summers et al., 2004; Ramsay and Wilga, 2007; Cappetta, 2012; Shimada, 2012; Crofts and Summers, 2014; Kolmann et al., 2015; Amadori et al., 2020a; Hamm, 2020). Durophagous teeth can greatly vary in occlusal morphologies, also exhibiting various degrees of convexity or a range of different cusps (Summers et al., 2004; Cappetta, 2012; Shimada, 2012; Crofts, 2015; Amadori et al., 2019b, 2020a). Additionally, various patterns of tooth abrasion are commonly associated with hard-shelled prey crushing diets in extinct and extant elasmobranchs (e.g. Claeson et al., 2010a; Summers et al., 2004; Amadori et al., 2019b, 2020a, b; Villalobos-Segura et al., 2019).

#### 8.3.1 Tooth shape implications

Occlusal cusps in molariform teeth of marine vertebrates (e.g. durophagous sharks) can be used to grab shelled prey, even concentrating the bite forces and increase stress on their shells during biting action (Summers et al., 2004; Shimada, 2012; Crofts and Summers, 2014; Crofts, 2015). Differently from what was hypothesized previously for the sharp-tipped crowns of †*Ptychodus mortoni*, the blunt apices of the cuspidate teeth in †*P. maghrebianus* sp. nov. (e.g. Fig. 6B) probably did not allow such a firm grip on the prey. In †*P. maghrebianus* sp. nov., the main function of molariform teeth was seemingly limited to transmitting the necessary compressive force for processing prey items. However, the balance between the ability to crush shelled prey and avoid possible crown breakage during the food processing can be of utmost importance in durophagous dentitions (see also Crofts, 2015). Although convex or cuspidate crowns require relatively less bite-force than flat teeth to crush rounded and/or coiled shells, the risk of crown breakage, due to a greater bite-strain, is higher in cuspidate teeth (Crofts and Summers, 2014; Crofts, 2015). In cuspidate toothed species, such as †*P. maghrebianus* sp. nov. and †*P. mortoni*, the lower bite-force required to crush the shells of the prey could compensate for a greater likelihood of occlusal surface fractures. Well-developed and narrow cusps are more effective in processing prey by reducing the affected area and simultaneously increasing the stress on the shell (Crofts and Summers, 2014; Crofts, 2015). In this perspective, the sharp and narrow cusps of †*P. mortoni* (Data Archiving Statement, below) could guarantee even greater bite performance. Moreover, inclined structures distribute the horizontal portion of the crush-load generally, which is, however, rarely sufficient to cause serious fractures (Tao et al., 1993). Resulting distortions or damages are therefore more concentrated outside the ‘core area’ of these structures (Tao et al., 1993). The oblique sides in cuspidate dental morphologies can mimic the load distribution in inclined structures by laterally shifting parts of the bite-load outside the cusp apex (core area). The tooth wear pattern on the cuspidate teeth of †*P. maghrebianus* sp. nov. that is mainly limited to the marginal areas (see Figs. 6–10) suggests a minimal involvement of the cusp apex in prolonged prey processing. The marginal areas of the cusp, conversely, experienced intense wear due to friction and scratching with prey items, which weakened it, further maximizing the bite stress in that area. An assumed interlocking pattern of upper and lower teeth during jaw closure (see above) would perfectly agree with such a scenario and the wear patterns found.

A multifarious function (crushing/grasping) and a consequent more intense use of the crown apex in species with narrower and/or sharper cusps (e.g. †*P. altior* and †*P. mortoni*) would lead to increased wear on the tooth tips (see also Shimada, 2012; Amadori et al., 2019b). The radial ornamentation pattern typical for teeth of both †*P. mortoni* and †*P. maghrebianus* sp. nov., can also affect the strength of the tooth crown, contrasting the bite stresses on the occlusal surface (see also Shimada, 2012). The highest degree of dental wear usually involves the occlusal surface in low-crowned durophagous elasmobranchs (e.g. †*Ptychodus latissimus*, †*P. mediterraneus* and *Rhinoptera bonasus*; see Summers, 2000; Amadori et al., 2019a, 2020a, b). These ‘hard-shelled prey specialists’ actually lack occlusal cusps completely but rather have relatively flat teeth that often are almost completely abraded (e.g. Summers, 2000; Kolmann et al., 2015; Amadori et al., 2019a, 2020a, b).

#### 8.3.2 Feeding apparatus implications

In numerous durophagous elasmobranchs (e.g. heterodontids, narcinids, ptychodontids and sphyrnids), the synergy between dental morphologies and tooth arrangement can have a direct influence on the evolution of trophic strategies and on the effectiveness of prey processing (e.g. crushing abilities; Cappetta, 1986; Wilga and Motta, 2000; Summers et al., 2004; Dean et al., 2008; Amadori et al., 2020a). Specifically, stresses and strains during prey processing can be distributed over multiple teeth that are closely interlocked with each other (e.g. imbricated dentitions), redirecting the bite-loads to specific areas of the dentition and improving their overall stability (Nobiling, 1977; Ramsay and Wilga, 2007; Huber et al., 2009; Shimada, 2012; Crofts, 2015). Moreover, cuspidate teeth of extant and extinct elasmobranchs can be extremely useful in grasping prey (Summers et al., 2004; Ramsay and Wilga, 2007; Dean et al., 2008; Huber et al., 2009; Shimada et al., 2009; Amadori et al., 2019b; Amini et al., 2020). The relevance of dental cusps (e.g. grasping dentitions) in prey-capture strategies for some durophagous elasmobranchs is widely documented (e.g. Wilga and Motta, 2000; Summers et al., 2004; Cappetta, 2012; Amadori et al., 2019b). For instance, *Heterodontus francisci* and *Sphyrna tiburo* use a composite dentition consisting of cuspidate anterior teeth used for grasping and latero-posterior molariform teeth suitable for crushing prey (e.g. crabs and shrimps; Wilga and Motta, 2000; Summers et al., 2004). For some of these ‘hard-shelled prey specialists’ the high cusps in the central part of their dentition could play a decisive role also in prey processing/manipulation. Among ptychodontids, †*Ptychodus mortoni* could simultaneously lock the shells and concentrate the force needed to crush them with the help of the cuspidate dental elements arranged along its entire feeding apparatus (Shimada, 2012). The vertical position of its dental cusps arranged within the dental plates (see Shimada, 2012; Fig. 6) consequently would increase and concentrated the pressure exerted by each tooth on the prey hard-tissue similar to the action of a peening tool (see also Frazzetta, 1988; Powlik, 1995; Evans and Sanson, 2003; Dean et al., 2008).

The blunt cusps described herein for the dentition of †*P. maghrebianus* sp. nov. (e.g. Fig. 6B) are not principally suitable for grabbing and holding prey (see also above). Nevertheless, the imbricated dentitions characterizing both species (†*P. maghrebianus* sp. nov. and †*P. mortoni*) could easily distribute the bite-load over the entire dental plate and reducing damage to individual crown surfaces (e.g. tooth wear; see also Huber et al., 2009; Shimada, 2012; Crofts, 2015). Furthermore, the combination of the mesio-distally curved lower tooth plate and the rounded cusp apices arranged along the central tooth rows in †*P. maghrebianus* sp. nov., might have allowed laterally directed mastication and subsequently an effective squelching of prey items. Accordingly, after an initial, coarse crushing of the shells using the cusped teeth (e.g. Figs. 6 and 7), the prey items subsequently would be shifted laterally for finer grinding with the help of the laterally positioned flat teeth (e.g. Fig. 10; see also Amadori et al., 2020a). Teeth with flat occlusal surfaces can more evenly distribute the applied load on shelled elements if more force for shell crushing is required. Flat teeth are therefore more suitable for fine shredding (see also Fossa-Mancini, 1921; Ford et al., 2009; Crofts, 2015). This crushing strategy would then explain the greater distal wear of lower teeth in †*P. maghrebianus* sp. nov (see Fig. 10) compared to greater tooth abrasions of the medial area within the dentitions of un-cuspidate species, such as †*P. mediterraneus* (see Amadori et al., 2020a, Fig. 19E). The mesiodistal displacement of the shells was probably arduous in taxa with teeth suitable for fixing prey items (e.g. †*P. mortoni*; see Shimada, 2012). However, doubts remain as to how many low-crowned durophagous specialists (e.g. *Aetomylaeus bovinus*, *Myliobatis californica*, †*Ptychodus mediterraneus* and *Rhinoptera bonasus*) concentrate prey processing in the medial area of their dental plates (see also Summers, 2000; Kolmann et al., 2015; Amadori et al., 2020a). Despite tooth plates having different degrees of mesiodistal curvature occur in a wide range of un-cuspidate durophagous predators, they normally avoid the lateral shifting of prey items (e.g. Summers, 2000; Cappetta, 2012; Kolmann et al., 2015; Amadori et al., 2020a). In low-crowned taxa, the absence of high cusps present in the central area of the dentition and a very slight occlusal curvature of their dental plates are perhaps sufficient to ensure the ‘central stay’ of the shell items once fixed between the dentitions. Additionally, some of them may have developed different crushing mechanisms, perhaps involving jaw movements, useful to concentrate/manipulate prey items at the center of the dental plate.

#### 8.3.3 Tooth replacement implications

Elasmobranch dentitions represent a peculiar object for studying the evolution of teeth in which a wide range of functionally demanding diets and the ability to continuously renew teeth during lifetime coexist (see Botella et al., 2009; Cappetta, 2012; Maisey et al., 2013; Belleggia et al., 2014; Underwood et al., 2015; Amadori et al., 2020a; Amini et al., 2020). The regular and continuous tooth replacement pattern (poly-phyodont) allows elasmobranchs to preserve a constant functionality and effectiveness of their dentitions (see Cappetta, 2012; Crofts and Summers, 2014; Amadori et al., 2020a; Amini et al., 2020). In some species, tooth renewal can change during ontogeny with juveniles usually being characterized by a faster replacement rate than that observed in adults (e.g. Wass, 1973; Reif et al., 1978). A slow dental renewal would involve a longer presence of teeth that become gradually abraded, damaged, or blunted and that, probably, are no longer able to fully perform their function within the feeding apparatus (see Amini et al., 2020). The ability to maintain functional tooth cusps despite an increased risk of bluntness in high-crowned dentitions may result in a crucial evolutionary advantage (see also Crofts and Summers, 2014; Crofts, 2015; Amini et al., 2020). Especially for ‘hard-shelled prey specialists’ having cuspidate teeth in combination with molariform dental elements could allow to exploit new trophic niches that are not available for other, possible competitors (Wilga and Motta, 2000; Shimada et al., 2009; Amadori et al., 2019b, Amini et al., 2020). A gradual, but noticeable, variation in the wear area of crown surface and/or abrasion intensity between newly formed and functional teeth can be related to different rates of tooth replacement in fossil sharks (e.g. Botella et al., 2009; Amadori et al., 2020a). Relatively slower tooth replacement rates were hypothesized in various extinct Palaeozoic taxa such as †*Cladoselache*, †*Ctenacanthus*, †*Doliodus*, †*Leonodus* and †*Pucapampella* (Williams, 2001; Botella et al., 2009; Maisey et al., 2013). In †*Ptychodus*, tooth wear resulting from the durophagous feeding habit can cover a wide range of wear patterns and abrasion intensity in species with both low- and high-crowned teeth (e.g. Woodward, 1912; Shimada, 2012; Amadori et al., 2019a, b, 2020a, b; Hamm, 2020). However, various aspects of the tooth-renewal ability in †*Ptychodus*, as well as in elasmobranchs in general, still are ambiguous and widely debated (see Belleggia et al., 2014; Cappetta, 2012; Underwood et al., 2015; Amadori et al., 2020a).

In †*Ptychodus maghrebianus* sp. nov., worn teeth are mainly found in the anterior portion of the lower dental plate, reaching the distal extremity of the dentition (see Fig. 5). Moreover, tooth wear mostly is faint and never involves the entire occlusal surface but is mainly limited to the lateral areas of the crown or of the cusp (see Figs. 6–10). These dental wear patterns indicate a rapid renewal capability of teeth, as already suggested for other cuspidate species (e.g. †*P. rugosus*; see Amadori et al., 2020a). However, in †*P. maghrebianus* sp. nov., the occlusal wear on teeth of the central rows is mitigated by the cuspidate shape (see above). Although a quick dental replacement remains the most likely hypothesis for high-crowned species, the lower degree of tooth wear in cuspidate †*Ptychodus* (e.g. †*P. maghrebianus* sp. nov.) compared to that in low-crowned taxa could be the result of different prey processing. A slow tooth renewal for †*P. maghrebianus* sp. nov. cannot therefore be ruled out a priori.

### 8.4 Diet and trophic hierarchies of the Asfla shark fauna during the Turonian

Although sharks are commonly considered top predators in extant marine communities, they can occupy very different trophic level, also having a wide range of feeding preferences (Cortés, 1999; Jacobsen and Bennett, 2013). Diet preferences including thin-shelled molluscs, decapod crustaceans and, occasionally, small fishes were more likely targeted prey for durophagous taxa, such as extant batoids and cuspidate ptychodontids (e.g. †*Ptychodus altior*, †*P. mortoni* and †*P. occidentalis*; see Shimada et al., 2009; Shimada, 2012; Everhart, 2017; Amadori et al., 2019b). Therefore, there is a clear correlation between durophagous dental morphologies and the ability of ‘hard-prey specialists’ to reach the bite-force necessary to break the shells of prey items (e.g. Crofts and Summers, 2014). Unfortunately, studies on the breaking load of prey items focused mainly on hard- and thick-shelled gastropods so far, which appear to require less bite-force, when subjected to be processed by high teeth with narrow cusps (Crofts and Summers, 2014). Assuming the same crushing patterns on gastropods for the shelled prey of †*Ptychodus maghrebianus* sp. nov., its large, rounded cusps could thus represent a disadvantage in food processing compared to narrower and more pointed teeth of other cuspidate species. Consequently, further studies on a larger and more varied group of prey items aimed at increasing our knowledge of the crushing patterns of shelled items are therefore mandatory. We assume that †*P. maghrebianus* sp. nov. had a diet comparable to other generalist †*Ptychodus* species with rounded cusps (e.g. †*P. altior* and †*P. occidentalis*) mainly consisting of thin-shelled molluscs and crustaceans (see also Shimada et al., 2009; Amadori et al., 2019b, 2020b). Furthermore, the possibility of preying on small fishes, as already suggested for †*P. occidentalis* (see also Shimada et al., 2009), is not excluded here for †*P. maghrebianus* sp. nov.

Durophagous batoids, such as the thornback ray †*Tingitanius* (platyrhinid), the sawfish †*Asflapristis* and †*Ptychotrygon* (sclerorhynchids) and possible guitarfishes (?*Rhinobatos*) were also part of the shark fauna from the lower Turonian (“*Mammites* bioevent”) of the Akrabou Formation (Goulmima region; Claeson et al., 2013; Villalobos-Segura et al., 2019, 2021). Although no evidence is available on the dietary preferences of the flat-toothed †*Tingitanius*, it probably had similar feeding preferences as those observed for its extant relatives. For instance, juveniles of *Platyrhina tangi* having almost flat teeth feed mostly on thin-shelled crustaceans (shrimps and mysids; see Yamaguchi et al., 2012). The almost flat, highly ornamented teeth and the robust jaws of †*Asflapristis*, together with considerable dental wear patterns, suggest a durophagous feeding habit for this Upper Cretaceous sawfish; pyramidal teeth of †*Ptychotrygon* were certainly suitable to crush shelled prey as well (Villalobos-Segura et al., 2019, 2021). Based on the poor swimming skills of the sclerorhynchids, the diet of †*Asflapristis* and †*Ptychotrygon* probably included bivalves and slow-moving prey, such as strongly armoured ammonites, whose shell could in fact be easily crushed by the flat as well as pyramidal and ornamented teeth of these extinct sawfishes (Cappetta, 1980; Villalobos-Segura et al., 2019, 2021). Small teeth with conical, pointed cusps belonging to the genus *Cretomanta* have been also found in the lower Turonian of Akrabou Formation (Goulmima region). *Cretomanta* was most probably a planktivorous elasmobranch putatively assigned to the family Aquilolamnidae (see Case et al., 1990; Villalobos-Segura et al., 2019; Vullo et al., 2021).

**Fig. 21:**
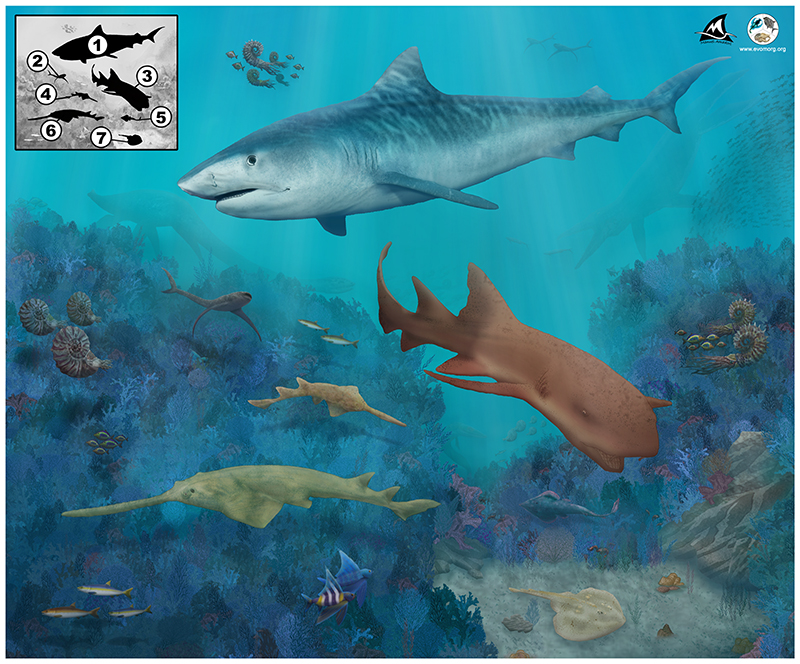
Interpretative palaeoenvironmental reconstruction of the Upper Cretaceous of Asfla area (Goulmima region; based on the relevant literature) with the main elasmobranch group hitherto reported (1, †*Squalicorax*; 2, †*Cretomanta*; 3, †*Ptychodus*; 4 †*Ptychotrygon*; 5,?*Rhinobatos*; 6, †*Asflapristis*; 7, †*Tingitanius*). Interpretative drawings are mostly speculative.

Among the Turonian durophagous predators of the Asfla area (Fig. 21), †*Ptychodus maghrebianus* sp. nov., the platyrhinid †*Tingitanius* and guitarfishes most likely represented second-level consumers, whose diet largely was based on thin-shelled molluscs, crustaceans and small fishes (see also Basusta et al., 2007; Lara-Mendoza et al., 2015). Based on the feeding niches of aquilopelagic (e.g., *Manta*) and macroceanic-tachypelagic (e.g., *Megachasma* and *Rhincodon*) planktivorous elasmobranchs (see Cort’es, 1999; Couturier et al., 2013), a similar trophic level (3–3.5 = second-level consumer) would be hypothesizable for *Cretomanta*. The probable ammonite hunters †*Asflapristis* and †*Ptychotrygon* (sclerorhynchid sawfishes) can be instead considered third-level predators. Generalist elasmobranchs (predators/scavengers), such as †*Squalicorax*, that were able to feed on an even larger prey (e.g., marine reptiles and other sharks) conversely occupied the top of the trophic hierarchies (‘apex predators’; see also Stewart, 1980; Schwimmer et al., 1997; Everhart and Caggiano, 2004; Gale et al., 2017; Ehret and Harrell, 2018; Amadori et al., 2020a; Cooper and Martill, 2020). The early Turonian marine environment of the Goulmima/Asfla region (Fig. 21) hosted a large array of teleosts including relatively small crossognathiforms (e.g. †*Goulmimichthys* and †*Kradimus*), varied shelled prey including bivalves (e.g. †*Astarte* and †*Rhynchostreon*), possible crustaceans and slow, strongly ornamented ammonite (e.g. †*Mammites nodosoides* and †*Thomasites rollandi*; Ettachfini and Andreu, 2004; Kennedy et al., 2008; Cavin et al., 2010; Lebedel et al., 2015; Meister et al., 2017; Cooper and Martill, 2020; Veysey et al., 2020), which all provided abundant food resources for lower level predators.

## 9 Conclusions

The new species, †*Ptychodus maghrebianus* sp. nov., from the lower Turonian of the Asfla area (Goulmima region, Morocco) is the third cuspidate ptychodontid species known with teeth that are characterized by radial occlusal ornamentations as in †*P. mortoni* Mantell (1836) and †*P. cyclodontis* Mutter et al. (2005). Qualitative and quantitative analyses performed on articulated and associated tooth sets in this study indicate a strong and statistically significant separation between †*P. mortoni* and †*P. maghrebianus* sp. nov. based on dental features. Although the heterodont dentition of †*P. maghrebianus* sp. nov. strongly differs from those described for the homodont †*P. cyclodontis*, further in-depth studies on the latter are desirable to clarify the systematic relationships between the three species.

Dental abrasions on the crushing plate of †*P. maghrebianus* sp. nov. are not compliant with the medial wear patterns typically documented for extinct and extant durophagous elasmobranchs equipped with tooth plates (batoids and ptychodontids). Although certainly suitable for crushing thin-shelled molluscs and crustaceans (durophagy), the dentition of †*P. maghrebianus* sp. nov. displays wear patterns that are markedly concentrated on the distal areas of the dental plate. The greater involvement of the small, flat, lateral teeth hypothesized for this second-level predator could be the first evidence of a peculiar and unexpected crushing strategy. Future detailed analyses of its dental histologies (e.g. orientation and thickness of the enameloid) and micro-wear patterns will provide further data and valuable insights on the mechanisms of prey processing for this and other durophagous sharks.

## Supplementary Material

F1

F2

F3

F4

F5

F6

Spreadsheet 1

Spreadsheet 2

Spreadsheet 3

Spreadsheet 4

Spreadsheet 5

Spreadsheet 6

Spreadsheet 7

Spreadsheet 8

## Figures and Tables

**Table 1:** List of nine specimens belonging to two cuspidate species of †*Ptychodus*, including the holotype of †*P. maghrebianus* presented herein and the selected comparison material assigned to †*Ptychodus* sp. and †*P. mortoni*. H, Holotype.

Described material:				
Taxa	Specimen	Type	N. of selected teeth	Provenience
†*P. maghrebianus* (**MAG**)**Comparison material:**	PIMUZ A/I 5056	Articulated tooth set (H)	90	Asfla area, Goulmima region, Errachidia Province, Morocco
**Taxa**	**Specimen**	**Type**	**N. of selected teeth**	**Provenience**
†*P. mortoni* (**MOR**)	NHMUK OR 28394	Isolated tooth (H)	1	Wilcox County, Alabama, USA
	FHSM VP-17415C	Associated tooth set	1	Jewell County, Kansas, USA
	FHSM VP-335	Associated tooth set	12	Scott County, Kansas, USA
	KUVP 119675	Articulated tooth set	2	Gove County, Kansas, USA
	ROM 21705	Articulated tooth set	90	Gove County, Kansas, USA
	UNSM 1194	Articulated tooth set	74	Lane County, Kansas, USA
†*Ptychodus* sp. (IND)	MT-G-011	Isolated tooth	1	Chejende’, Trujillo, Venezuela
	MT-G-012	Isolated tooth	1	Chejende’, Trujillo, Venezuela
	MT-G-013	Isolated tooth	1	Chejende’, Trujillo, Venezuela

**Table 2:** Tooth measurements in millimeters of lower dental plate of †*Ptychodus maghrebianus*. P, placement within the dental plate (see interpretative line drawing in Fig. 3A-C); CW, Crown width; CL, Crown length; CH, Crown height. Parentheses indicate estimated value.

P	CW min.	CW max.	CL min.	CL max.	CH min.	CH max.
m	~30	~30	~ (19)	~21	~16	~17
l1	~ (24)	~ (26)	~16	~17	~9	~ (13)
l2	~19	~22	~12	~ (14)	~ (7)	~ (10)
l3	~17	~19	~10	~12	~5	~ (6)
l4	~ (15)	~17	~9	~10	~4	~ (5)
l5	~14	~ (15)	~8	~10	~4	~ (5)
l6	~14	~16	~8	~9	~3	~3
l7	~ (13)	~ (14)	~6	~7	~ (2)	~ (2)
l8	~13	~13	~6	~6	~3	~3
l9	~12	~ (13)	~5	~5	~2	~ (3)
l10	~10	~ (11)	~4	~4	~2	~2
l11	~9	~9	~3	~3	~2	~2

**Table 3:** Nonparametric tests (PERMANOVA and ANOSIM) used to assess significant differences in morphospace occupation between the two species †*Ptychodus maghrebianus* (MAG) and †*P. mortoni* (MOR) and three indeterminate teeth (IND). The significance is computed by 9999 permutations of group membership. Euclidean distance is the chosen measurement unit. The overall p-value for both PERMANOVA and ANOSIM is 0.0001. Asterisks indicate significant comparisons (p < 0.05) thereby suggesting that groups exhibit considerably different morphospace occupation.

PERMANOVA		
p-values	MOR	IND
**MAG**	0.0003*	0.0003*
**MOR**		0.1293
**F-values**	**MOR**	**IND**
**MAG**	98.0200	14.0900
**MOR**		2.7520
**ANOSIM**		
**p-values**	**MOR**	**IND**
**MAG**	0.0003*	0.0030*
**MOR**		0.4446
**R-values**	**MOR**	**IND**
**MAG**	0.4010	0.7637
**MOR**		0.1834

**Table 4:** Canonical variate analysis results of comparisons between the two species †*Ptychodus maghrebianus* (MAG) and †*P. mortoni* (MOR) and three indeterminate teeth (IND). The significance is computed by 9999 permutations of group membership. Asterisks indicate significant comparisons (p < 0.05) thereby suggesting that groups exhibit considerably different morphospace occupation

**Hotelling’s *t*-test**		
p-values	MOR	IND
**MAG**	0,00000000001*	0,00000063385*
**MOR**		0,00000001223*
**Squared Mahalanobis distance**		
**p-values**	**MOR**	**IND**
**MAG**	2,3510	47,7670
**MOR**		41,5840

**Table 5:** Classification (Jackknifed) resulting from the comparison (CVA; see Fig. 17) between †*Ptychodus maghrebianus* (MAG), †*P. mortoni* (MOR) and three indeterminate teeth (IND). 93.15% of the teeth has been correctly classified.

Groups	MAG	MOR	IND	Total
**MAG**	87	3	0	90
**MOR**	1	144	10	155
**IND**	0	3	0	3
**Total**	88	150	10	248
