# Supplementary figures and images for: A new cuspidate ptychodontid shark (Chondrichthyes; Elasmobranchii), from the Upper Cretaceous of Morocco with comments on tooth functionalities and replacement patterns
Source: J Afr Earth Sci. Author manuscript; Available in PMC 2022 Mar 1. (PMC7612291; doi:10.1016/j.jafrearsci.2021.104440)

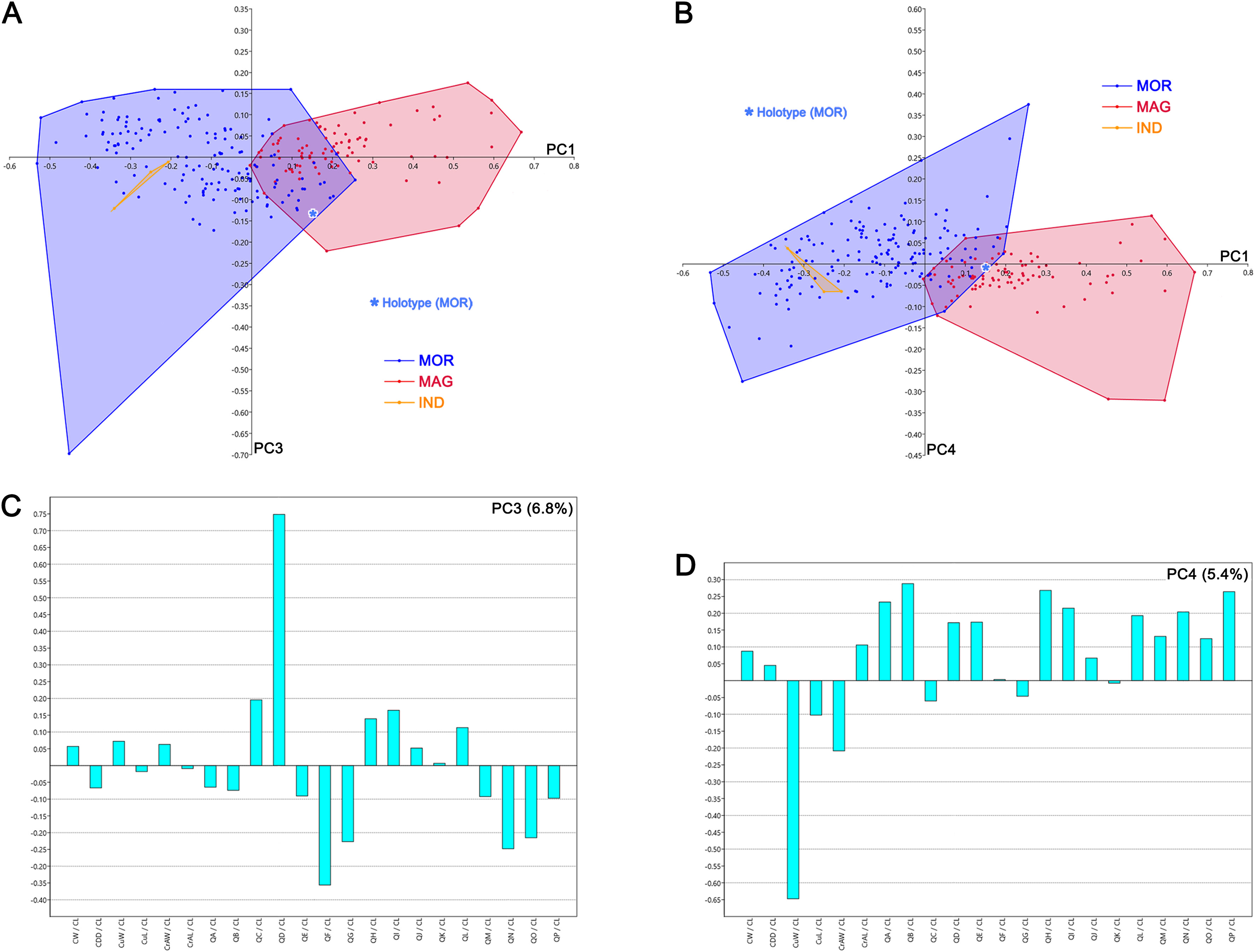

Supplement: F1 [file EMS140888-supplement-F1.jpg]

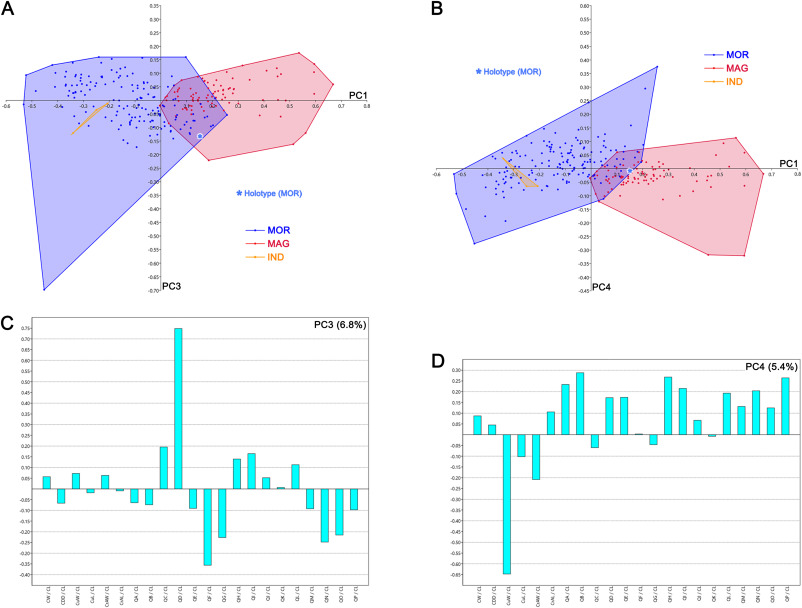

Supplement: F2 [file EMS140888-supplement-F2.jpg]

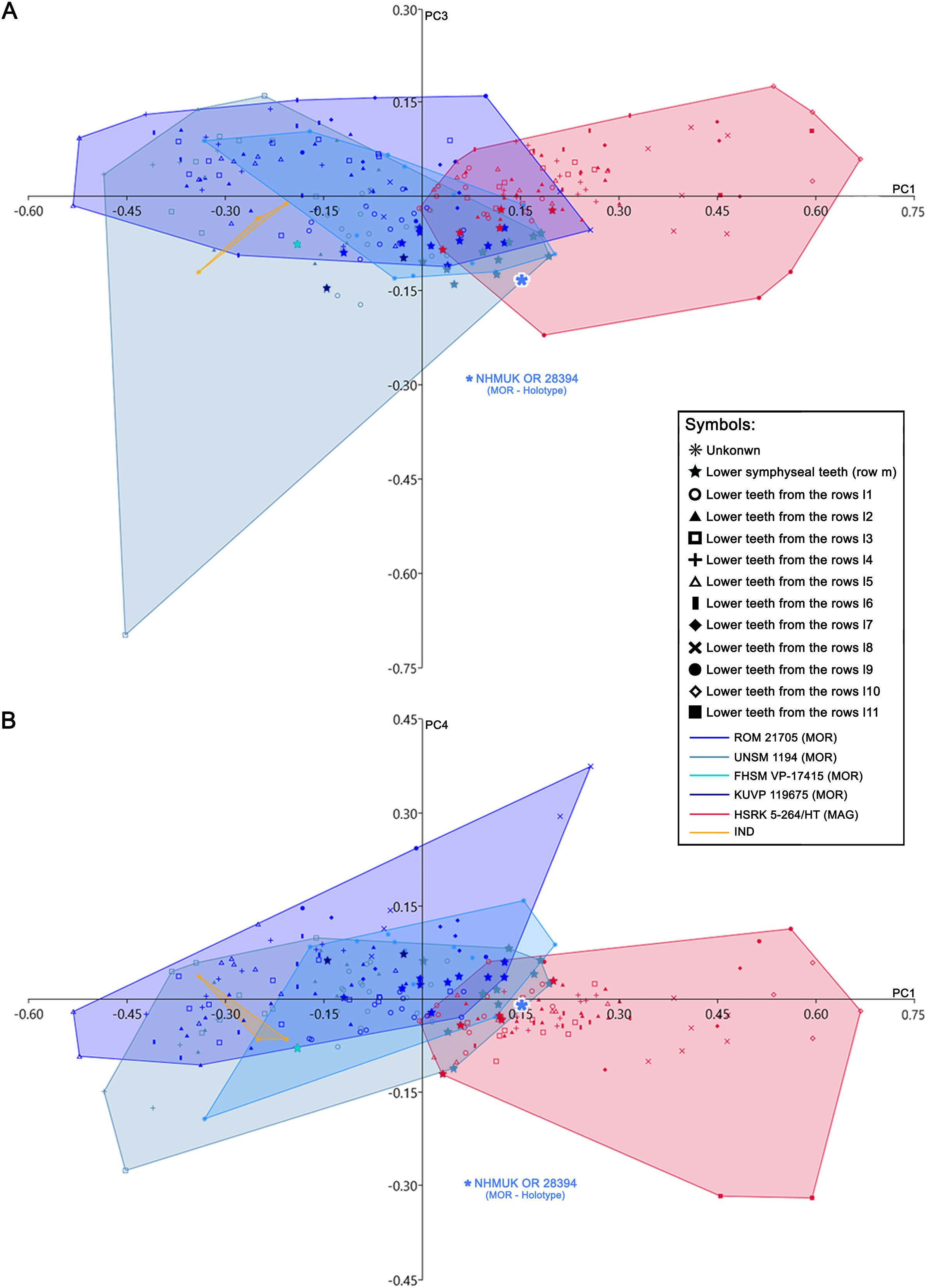

Supplement: F3 [file EMS140888-supplement-F3.jpg]

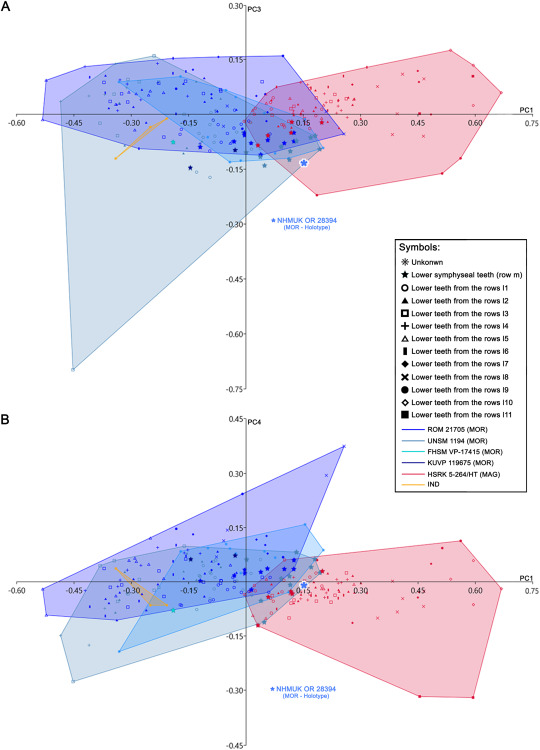

Supplement: F4 [file EMS140888-supplement-F4.jpg]

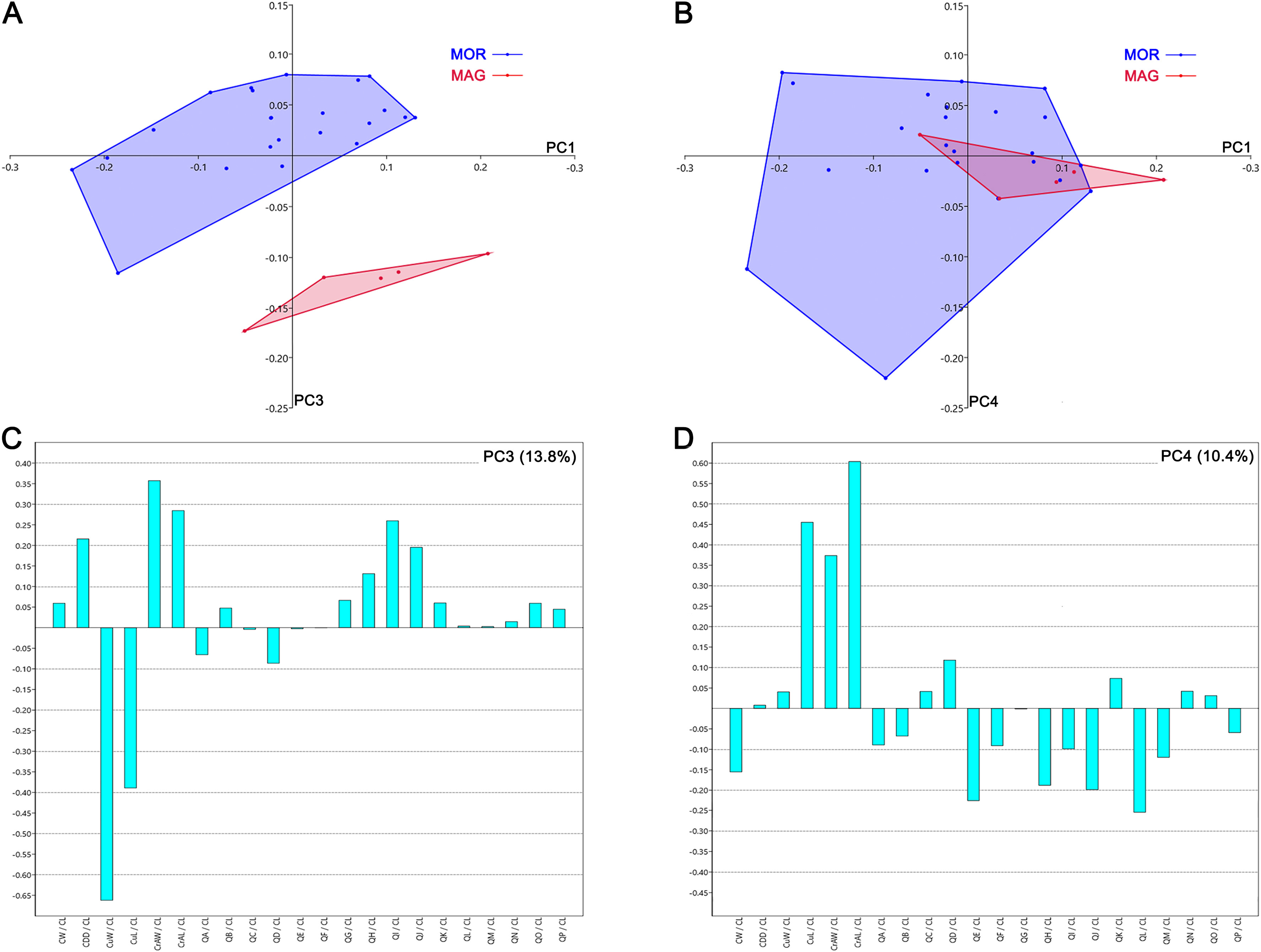

Supplement: F5 [file EMS140888-supplement-F5.jpg]

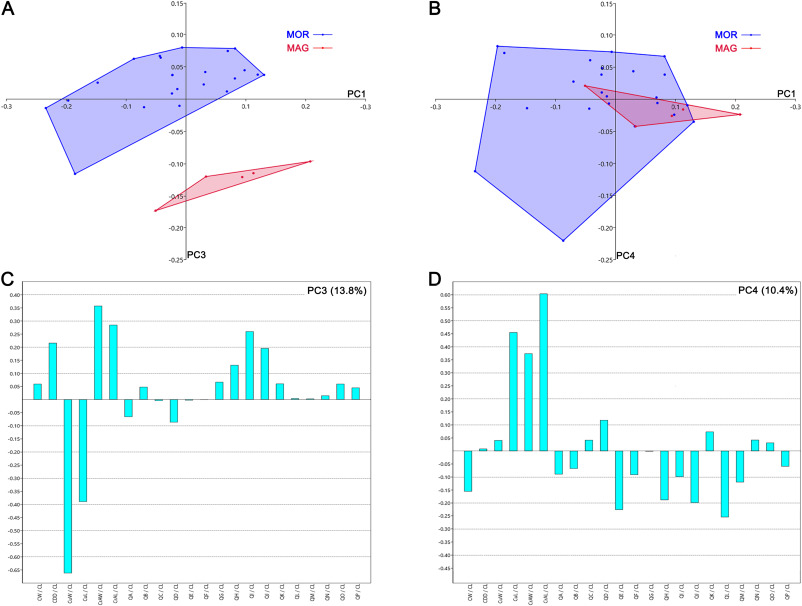

Supplement: F6 [file EMS140888-supplement-F6.jpg]
